# Supplementary material for: A family of small proteins links c-di-GMP riboswitch signaling with sporulation in Clostridioides difficile
Source: Nat Commun. 2026 Oct 4;17:11319. doi: 10.1038/s41467-026-78452-6 (PMC13724677; doi:10.1038/s41467-026-78452-6)
Supplement: Supplementary file 2 [file 41467_2026_78452_MOESM11_ESM.pdf]

nature portfolio

Peer Review File

A family of small proteins links c-di-GMP riboswitch signaling with sporulation in Clostridioides difficile

Corresponding Author: Dr Johann Peltier

This file contains all reviewer reports in order by version, followed by all author rebuttals in order by version.

Version 0:

Reviewer comments:

Reviewer #1

(Remarks to the Author)
This study describes the discovery of a new mechanism of regulation of spore development in C. difficile. Two prior studies had reported that the signaling molecule c-di-GMP negatively regulates C. difficile sporulation, which is important given that spore development is necessary for transmission of this pathogen between hosts. However, neither study determined the underlying mechanism. Here, the authors identify a set of small proteins whose production is negatively regulated by c-di-GMP via riboswitches and which are critical for proper spore formation. This work is exciting because it (1) determined how c-di-GMP inhibits C. difficile spore development, (2) identified new targets of c-di-GMP regulation in C. difficile, and (3) revealed a novel mechanism of sporulation regulation. As such, the work represents a substantial advance in the field. The methodology is sound, and experiments are rigorously performed; replicates, controls, and statistical analyses are appropriate. The results are clearly presented, and the manuscript is well-written.

General comments:
1. How conserved is this mechanism? The gene paralogs are conserved, but do these proteins, particularly CD1980.2, function similarly in clinically-relevant strains that have higher sporulation efficiency?

2. This work suggests that c-di-GMP levels change during sporulation to modulate gene expression. Is there evidence that c-di-GMP decreases during sporulation to stimulate transcription of CD1980.2 and/or its paralogs? While measuring c-di-GMP directly would likely be challenging, analyzing expression of CD1980.2 (or other c-di-GMP-regulated gene) during sporulation would be a viable proxy.

Minor comments:

CD1419, encoded adjacent to CD1420, was found to be induced upon dccA expression. This was interpreted as suggesting the genes are co-transcribed. While this may be the case, the current data only support the conclusion that dccA expression, and thereby c-di-GMP, promote CD1419 transcription. The mapped reads from RNA-seq may be informative regarding transcriptional readthrough between these genes.

In Figure 2C, the dark colors chosen make the black text difficult to read. Recommend using lighter colors or white text.

In Fig 4D, the “WT” labels are not needed (and inaccurate since the strain contains the reporter).

L303-305, it is somewhat unclear from the wording whether CD1667.1 or CD1980.2 promotes hypersporulation.

L717, correct SpoA to Spo0A.

Reviewer #2

(Remarks to the Author)

This well-written manuscript by Lobo, et al. describes the discovery of a small family of membrane-associated proteins that are directly repressed in response to high intracellular levels of c-di-GMP. This decrease in their expression and accumulation resulted in decreased C. difficile spore formation. Additional RNA-seq analysis revealed that these proteins positively influence sporulation initiation through an unknown mechanism. Overexpression of a single member resulted in hypersporulation, while deletion of these proteins decreased sporulation in an additive manner. Discovery of this family of small proteins adds an additional regulatory connection between c-di-GMP levels and sporulation frequency that has broad interest to the Clostridial and sporulation fields. Additional data supporting this connection would strengthen the conclusions.

Major concern:
Fig 6B: Because there is a possibility that very low sporulation frequency in the Δ7 mutant masks the impact on sporulation when c-di-GMP is overproduced, there is a gap in the connection between high c-di-GMP levels mediating decreased sporulation through transcriptional regulation of these small proteins. Further, there is no complementation data restoring the low sporulation phenotype of the Δ7 strain. One approach to accomplish each of these tasks is to first overexpress the CD1980.2 construct in the Δ7 background in two ways: from its native promoter (with the riboswitch) and a constitutive promoter (or an inducible promoter that is not Ptet. These constructs would be predicted to result in increased sporulation in the Δ7 background, as this protein family is predicted to function redundantly. Next, demonstrate that the increase in sporulation is mediated by direct regulation via the c-di-GMP-dependent riboswitch by overexpressing Ptet-DccA to increase c-di-GMP levels. In high c-di-GMP conditions, sporulation should only decrease in the strain expressing CD1980.1 from its native transcriptional unit, not the one with a constitutive promoter. These data would provide a definitive connection between c-di-GMP levels, c-di-GMP-dependent regulation of this small family of proteins, and C. difficile sporulation frequency.

Minor comments:
If 1980/2309 make up 85+% of relative transcripts when c-di-GMP is induced, why is sporulation not as impacted between the WT strain and the Δ2 strain? Is this because of potential differences in expression of proteins between TY medium and sporulation agar or is there an alternative explanation?
Fig 5A: Generally, C. difficile sporulation does not continue significantly beyond 24 h on 70:30 agar. The observation that sporulation frequency more than doubled between 24 h and 72 h may be artificially higher because of vegetative cell lysis over time. Are the total number of vegetative cells and spores counted at each time point similar or does this number decrease over time? It would be clarifying to provide, in the supplemental, phase contrast micrographs of each time point to show the continued increase in sporulation frequency versus accumulated cell lysis of non-sporulating vegetative cells at 48 h and 72 h versus 24 h.
Fig. 6A: The statistical comparison should be between the Δ7 strain and the pPtet-dccA strain based on the results (lines 264-267), not between the WT and each mutant individually (could note as not significant, ns).
Line 312 - “near perfect” consensus site for CD2386.1
Line 317-319 – Because of the suggestion that c-di-GMP may affect RgaR activity, did the authors check to see if transcription of any other known RgaR targets were impacted by the overexpression of DccA?

Reviewer #3

(Remarks to the Author)
Manuscript ID: NCOMMS-25-94626

Manuscript Title: A family of small proteins links c-di-GMP riboswitch signaling with sporulation in Clostridioides difficile

01-09-2026
Author summary
The study by Lobo et al. identifies a conserved family of small, membrane-associated proteins that function as novel components linking c-di-GMP signaling to sporulation control in Clostridioides difficile. These genes are transcriptionally repressed by class I c-di-GMP–responsive riboswitches through premature termination. Overexpression of a single family member activates sporulation genes and enhances spore formation, whereas successive deletions of these genes lead to a stepwise decrease in sporulation efficiency, culminating in a severe defect when all family members are absent. Elevated intracellular c-di-GMP levels do not further inhibit sporulation in the complete deletion mutant, supporting the conclusion that c-di-GMP acts by repressing expression of these small-protein genes. Overall, this work establishes a mechanistic link between c-di-GMP signaling and developmental regulation, thereby broadening the known repertoire of bacterial second messenger outputs.

Review
This manuscript is well written and logically structured. The experimental design is sound, and the data presentation appears accurate and convincing. Overall, the study might be of broad interest to microbiologists interested in sec. messenger signaling, as it provides findings that connect c-di-GMP signaling to cellular differentiation processes in this case sporulation. This has been so far only reported for Streptomyces spec and Bacillus anthracis to my knowledge. Nevertheless, several major points should be addressed to further strengthen the manuscript and permit publication, as outlined below.

General/ major comments
1. Terminology in c-di-GMP/second messenger signaling
Putative binding of c-di-GMP to newly identified proteins?
The terms c-di-GMP effector should be reserved for molecules that directly bind c-di-GMP, such as PilZ-domain proteins, transcription factors, degenerated GGDEF/EAL domain proteins, or c-di-GMP-responsive riboswitch RNAs. The proteins themselves, reported in this study, are likely to not directly bind c-di-GMP. However, this is in my view discriminated in the manuscript. Please provide reasonable arguments why this has not been tested in the current study (see below: bioinformatic/ structural analysis of “small-membrane proteins”).

Mechanistically, the riboswitch represents the true c-di-GMP effector, whereas the encoded membrane proteins function downstream as targets or outputs of the signaling pathway.
To ensure accurate and precise terminology throughout the manuscript, I suggest to revise sentences that may imply direct binding of c-di-GMP by these proteins. Phrasing such as the following would be more appropriate:
“X is a c-di-GMP–regulated protein whose expression is controlled by a c-di-GMP–responsive riboswitch,” or
“X functions in a c-di-GMP signaling output pathway that is transcriptionally regulated via a c-di-GMP–responsive riboswitch.”
In particular, corresponding statements in the abstract and introduction (e.g., lines 25, 36, 90, and 94) should be adjusted to convey this distinction clearly and consistently.

2. Relation to previous transcriptional analyses (e.g., McKee et al., mSphere 2018)
The authors should clarify how their RNA-seq based approach differs from previous transcriptomic studies, such as that of McKee et al. (2018). Specifically, were genes CD1424, CD1990, CD2309, and CD3368 also identified in earlier datasets as strongly repressed under conditions of elevated c-di-GMP? It is not entirely clear whether the present transcriptomic analysis newly identified these small membrane-protein genes or confirmed prior observations with greater resolution. Providing a direct comparison to the earlier studies would help position this work within the existing literature and underscore the novelty of the findings, potentially.

3. Schematic overview of c-di-GMP signaling
I recommend to include a schematic overview illustrating c-di-GMP metabolism especially its regulatory outputs in Clostridioides difficile. This figure would effectively contextualize the newly identified c-di-GMP-responsive genes encoding small membrane proteins within the broader signaling network. Such a diagram could include additionally key known functions of c-di-GMP in C. difficile: riboswitch-mediated control of motility (flgB operon via class I riboswitches), toxin production, surface adhesion and type IV pilus assembly (pilA1 via class II riboswitch).

4. Bioinformatic/ structural analysis of small membrane proteins
In my view, the authors should complement their AA sequence alignments with secondary structure predictions and AlphaFold models, including superimpositions of homologs (maybe also from related species?). These analyses would provide readers with a clear visual representation of the proteins' predicted structures and conserved features. They could be presented as new panels in an existing figure (e.g., Figure 3) or as a supplementary figure.
I predicted the protein structure of CD2309 with alpha-fold, obviously it does predeict (more or less with high confidence) 3-4 a-helices arranged in a parallel manner. Please provide more information on the putative hydrophobic helix at the C-Terminus! Is the C-Terminus indeed critical for membrane insertion. I would suggest localization assays with truncated versions based on alpha-fold predictions!

Suggestion for an additional paragraph within the manuscript:
The small membrane proteins encoded by CD1424, CD1990, CD2309, and CD3368 in Clostridioides difficile strain 630 exhibit high sequence conservation, particularly in their predicted helical regions, as revealed by multiple sequence alignments. Secondary structure predictions using PSIPRED confirm 3-4 transmembrane alpha-helices in each protein, with the C-terminal helix displaying strong hydrophobicity suggestive of membrane anchoring. AlphaFold2 models for CD2309 demonstrate high-confidence predictions (pLDDT > 90 for core helices) of four parallel alpha-helices forming a compact bundle, consistent with the reviewer's observation and alignments with homologs from related Clostridia species such as Clostridium sporogenes.

5. Relevance of small membrane proteins during spore formation/ spore germination?
Small membrane proteins like those encoded by CD1424, CD1990, CD2309, and CD3368 show potential relevance to spore formation and germination due to their predicted transmembrane helical structures, which could facilitate signaling or structural roles at the membrane during sporulation stages. While direct localization via fluorescence microscopy poses challenges due to their small size and hydrophobicity, transcriptional analysis such as qRT-PCR during sporulation time courses (e.g., monitoring expression peaks at stages II-IV) offers a feasible, indirect way to link them to sporulation or germination processes.
In view of the substantial concerns detailed above, I regret to conclude that in my view the manuscript is most likely not suitable for publication in its present form and therefore must recommend major revision!
Kind regards

Version 1:

Reviewer comments:

Reviewer #1

(Remarks to the Author)
The authors were thoughtful and thorough in their responses to reviewer comments and revisions to the manuscript. The new data strengthen the conclusions.

Reviewer #2

(Remarks to the Author)
The authors spent considerable time, thought, and effort into responding to the reviews, and the manuscript is now ready for publication. This study fills an important gap in the understanding of c-di-GMP control of C. difficile sporulation and identifies an intriguing, novel family of proteins that should generate broad interest.

Reviewer #3

(Remarks to the Author)
Thank you for the thorough revision of your manuscript. You have carefully addressed the concerns raised in the initial review, and the additional data and clarifications have improved both the rigor and clarity of the study.

In particular, the revisions to the methodology and the expanded explanations of your data analysis strengthen the support for your conclusions. The manuscript is now more transparent and easier to follow, and the key findings are presented in a convincing manner.

I do not have further substantive comments at this stage and consider the work suitable for publication.

Due to a recent transition out of academia and limited availability, I am unfortunately unable to provide additional rounds of detailed review. I hope, however, that the current version can proceed toward publication.

Open Access This Peer Review File is licensed under a Creative Commons Attribution 4.0 International License, which permits use, sharing, adaptation, distribution and reproduction in any medium or format, as long as you give appropriate credit to the original author(s) and the source, provide a link to the Creative Commons license, and indicate if changes were made.
In cases where reviewers are anonymous, credit should be given to 'Anonymous Referee' and the source.
The images or other third party material in this Peer Review File are included in the article’s Creative Commons license, unless indicated otherwise in a credit line to the material. If material is not included in the article’s Creative Commons license and your intended use is not permitted by statutory regulation or exceeds the permitted use, you will need to obtain permission directly from the copyright holder.
To view a copy of this license, visit https://creativecommons.org/licenses/by/4.0/

Point-by-point response to reviewer comments

NCOMMS-25-94626: **A family of small proteins links c-di-GMP riboswitch signaling with sporulation in *Clostridioides difficile***

We sincerely thank the reviewers for their thoughtful and constructive evaluation of our work. We have addressed all comments in detail below and revised the manuscript accordingly. The suggested changes have materially strengthened the clarity, interpretation, and overall quality of the manuscript, and we appreciate the opportunity to revise it in response to the feedback. Reviewer comments are reproduced in full in black text and our responses are in blue text.

REVIEWER COMMENTS AND RESPONSES

Reviewer #1 (Remarks to the Author):

This study describes the discovery of a new mechanism of regulation of spore development in C. difficile. Two prior studies had reported that the signaling molecule c-di-GMP negatively regulates C. difficile sporulation, which is important given that spore development is necessary for transmission of this pathogen between hosts. However, neither study determined the underlying mechanism. Here, the authors identify a set of small proteins whose production is negatively regulated by c-di-GMP via riboswitches and which are critical for proper spore formation. This work is exciting because it (1) determined how c-di-GMP inhibits C. difficile spore development, (2) identified new targets of c-di-GMP regulation in C. difficile, and (3) revealed a novel mechanism of sporulation regulation. As such, the work represents a substantial advance in the field. The methodology is sound, and experiments are rigorously performed; replicates, controls, and statistical analyses are appropriate. The results are clearly presented, and the manuscript is well-written.

General comments:
1. How conserved is this mechanism? The gene paralogs are conserved, but do these proteins, particularly CD1980.2, function similarly in clinically-relevant strains that have higher sporulation efficiency?

We thank the reviewer for this important point. Our comparative genomic analyses indicate that the CD1980.2 family and several of its paralogs are broadly conserved among *C. difficile* lineages, including clinically relevant strains. For example, all seven family members are present in epidemic strains such as R20291 and UK1, suggesting that this regulatory module is widely conserved within the species.

To address the reviewer's question experimentally, we introduced a plasmid expressing CD1980.2 into the clinically relevant epidemic isolate UK1 and compared sporulation frequencies with a vector-only control. In contrast to strain 630Δ*erm*, *CD1980.2* overexpression did not further enhance sporulation in UK1 under the conditions tested. Notably, the UK1 vector control already exhibited a high sporulation efficiency (~18%), comparable to that observed in the *CD1980.2*-overexpressing 630Δ*erm* strain. These results

indicate that UK1 sporulates efficiently under these conditions and that additional *CD1980.2* expression does not further increase sporulation output.

One possible explanation is that the contribution of CD1980.2 becomes limiting in strain 630Δ*erm* but not in UK1, which may already express sufficient levels of this protein family or possess a regulatory network optimized for efficient sporulation. A more definitive assessment of conservation would require construction of multiple-gene deletion mutants in clinically relevant strains. However, given the presence of seven highly similar paralogs in UK1, such experiments are beyond the scope of the present study.

We have now included these new experimental data in the result section (new Supplementary Fig. 6A and lines 270 to 277).

2. This work suggests that c-di-GMP levels change during sporulation to modulate gene expression. Is there evidence that c-di-GMP decreases during sporulation to stimulate transcription of CD1980.2 and/or its paralogs? While measuring c-di-GMP directly would likely be challenging, analyzing expression of CD1980.2 (or other c-di-GMP-regulated gene) during sporulation would be a viable proxy.

We thank the reviewer for this insightful comment. Our study demonstrates that elevated c-di-GMP levels repress the expression of CD1980.2 and its paralogs and inhibit sporulation. This indicates that high c-di-GMP levels are incompatible with the expression of these genes and efficient spore formation. However, our data do not demonstrate that a decrease in c-di-GMP is required to trigger their expression, as these small proteins are already expressed during exponential growth under standard in vitro conditions, where c-di-GMP levels are presumed to be low. This suggests that these small proteins may act as permissive factors for sporulation, whose presence is required prior to entry into the developmental program, rather than being specifically induced during sporulation. Importantly, while overexpression of CD1980.2 significantly increased sporulation efficiency in 630Δ*erm*, this phenotype results from strong artificial expression and does not imply that increased expression of this protein is normally required during sporulation. Rather, it likely reflects amplification of a permissive function already provided at basal expression levels.

To directly address the reviewer's suggestion, we performed qRT-PCR analyses of the representative small-protein gene *CD2309* together with additional c-di-GMP riboswitch-controlled genes (*CD2831*, *pilA1*, and *ppep-1*) in the wild-type strain grown under sporulation conditions (70:30 medium). To independently evaluate the effects of growth stage and growth condition, gene expression was compared between early (4 h) and late (16 h) time points in liquid medium, and between liquid and solid (70:30 agar) cultures at 16 h. Under all conditions tested, we did not observe significant changes in expression for any of these genes. These results do not support the existence of large global changes in c-di-GMP signaling during sporulation. Instead, they are consistent with the current view that c-di-GMP signaling often relies on modest and spatially localized changes in intracellular concentration, generated by specific diguanylate cyclases and phosphodiesterases acting in local signaling modules, rather than on major changes in the total cellular c-di-GMP poo (PMID: **33640237**). Our data therefore suggest that c-di-GMP may contribute to sporulation control through such localized or subtle signaling events rather than through a large global change in intracellular c-di-GMP levels. We have now incorporated these new data into the revised Results section (lines 278–

284; Supplementary Fig. 6B-C) and discuss their implications in the Discussion (lines 358 to 368), where we propose that c-di-GMP-dependent control of sporulation may involve localized or subtle signaling events rather than large global changes in intracellular c-di-GMP levels.

Minor comments:

CD1419, encoded adjacent to CD1420, was found to be induced upon dccA expression. This was interpreted as suggesting the genes are co-transcribed. While this may be the case, the current data only support the conclusion that dccA expression, and thereby c-di-GMP, promote CD1419 transcription. The mapped reads from RNA-seq may be informative regarding transcriptional readthrough between these genes.

We thank the reviewer for this important comment. Upon re-examining the RNA-seq read coverage, we observed a clear drop in signal between *dccA* and CD1419, indicating the absence of transcriptional readthrough under our conditions. Furthermore, previously published genome-wide transcription start site mapping identified an independent promoter upstream of CD1419. We have therefore revised the manuscript to remove the statement suggesting co-transcription and now conclude that CD1419 is likely independently transcribed but strongly induced upon elevated c-di-GMP levels (lines 110 to 117). To facilitate evaluation of this region, we have also included the RNA-seq read coverage across the *dccA–CD1419* locus in new Supplementary Figure 1.

In Figure 2C, the dark colors chosen make the black text difficult to read. Recommend using lighter colors or white text.

We thank the reviewer for this suggestion. The color scheme in Fig. 2C has been modified to improve readability, using bold white text to ensure better contrast. We have also applied the same modifications to Supplementary Fig. 2A and Supplementary Fig. 3 A-C for consistency and improved clarity throughout the manuscript.

In Fig 4D, the “WT” labels are not needed (and inaccurate since the strain contains the reporter).

We thank the reviewer for this suggestion. The “WT” labels have been removed from Fig. 4D to avoid confusion, as the strain used carries the reporter construct.

L303-305, it is somewhat unclear from the wording whether CD1667.1 or CD1980.2 promotes hypersporulation.

We thank the reviewer for pointing out this ambiguity. The sentence has been revised to clarify that hypersporulation is specifically associated with CD1980.2, whereas CD1667.1 reduces sporulation despite their high sequence identity (lines 374 to 376).

L717, correct SpoA to Spo0A.

We thank the reviewer for noting this typographical error. SpoA has been corrected to Spo0A in the revised manuscript.

Reviewer #2 (Remarks to the Author):

This well-written manuscript by Lobo, et al. describes the discovery of a small family of membrane-associated proteins that are directly repressed in response to high intracellular levels of c-di-GMP. This decrease in their expression and accumulation resulted in decreased C. difficile spore formation. Additional RNA-seq analysis revealed that these proteins positively influence sporulation initiation through an unknown mechanism. Overexpression of a single member resulted in hypersporulation, while deletion of these proteins decreased sporulation in an additive manner. Discovery of this family of small proteins adds an additional regulatory connection between c-di-GMP levels and sporulation frequency that has broad interest to the Clostridial and sporulation fields. Additional data supporting this connection would strengthen the conclusions.

Major concern:
Fig 6B: Because there is a possibility that very low sporulation frequency in the Δ7 mutant masks the impact on sporulation when c-di-GMP is overproduced, there is a gap in the connection between high c-di-GMP levels mediating decreased sporulation through transcriptional regulation of these small proteins. Further, there is no complementation data restoring the low sporulation phenotype of the Δ7 strain. One approach to accomplish each of these tasks is to first overexpress the CD1980.2 construct in the Δ7 background in two ways: from its native promoter (with the riboswitch) and a constitutive promoter (or an inducible promoter that is not Ptet. These constructs would be predicted to result in increased sporulation in the Δ7 background, as this protein family is predicted to function redundantly. Next, demonstrate that the increase in sporulation is mediated by direct regulation via the c-di-GMP-dependent riboswitch by overexpressing Ptet-DccA to increase c-di-GMP levels. In high c-di-GMP conditions, sporulation should only decrease in the strain expressing CD1980.1 from its native transcriptional unit, not the one with a constitutive promoter. These data would provide a definitive connection between c-di-GMP levels, c-di-GMP-dependent regulation of this small family of proteins, and C. difficile sporulation frequency.

We thank the reviewer for this thoughtful and constructive suggestion. We agree that an epistasis experiment uncoupling expression of the small proteins from c-di-GMP-dependent riboswitch regulation would provide the most direct demonstration that c-di-GMP controls sporulation through this protein family.

First, in response to the reviewer's comment regarding complementation of the Δ7 mutant, we introduced a P_{tet}-*CD1980.2* construct into the Δ7 background. Expression of CD1980.2 restored sporulation in this background, demonstrating that the severe sporulation defect of the multiple-deletion mutant is directly attributable to loss of this small-protein family. These new data are now presented in Fig. 5E and described in the revised Results section (lines 295 to 298).

We also attempted to functionally separate CD1980.2 expression from its native riboswitch-dependent regulation. To this end, we generated constructs lacking the native riboswitch-containing 5′ regulatory region as suggested by the reviewer. Unexpectedly, these constructs failed to promote sporulation in the wild type backgrund, preventing a direct test of the epistasis model proposed by the reviewer. It is possible that the riboswitch-containing leader contributes to transcript stability and/or efficient expression of this unusually small gene.

As an alternative approach, we attempted to manipulate intracellular c-di-GMP levels through overexpression of the phosphodiesterase PdcB, which has previously been linked to sporulation regulation through reduction of c-di-GMP levels in the UK1 strain (PMID: 34606654). However, under our experimental conditions, PdcB overexpression did not measurably increase sporulation in strain 630Δ*erm*, preventing a meaningful epistasis analysis.

We therefore acknowledge that the definitive experiment proposed by the reviewer remains to be performed. Accordingly, we cannot formally exclude the possibility that the severe sporulation defect of the Δ7 mutant masks a residual contribution of elevated c-di-GMP levels. Nevertheless, several independent observations support a functional connection between c-di-GMP signaling, repression of this protein family, and sporulation: (i) elevated c-di-GMP strongly represses expression of all five riboswitch-associated genes, (ii) progressive deletion of family members results in progressively reduced sporulation, (iii) the Δ7 mutant displays a severe sporulation defect that is rescued by *CD1980.2* complementation, and (iv) ectopic expression of *CD1980.2* promotes sporulation. Together, these data support a model in which c-di-GMP inhibits sporulation, at least in part, through repression of this family of small proteins.

We now explicitly acknowledge in the Discussion section that future epistasis analyses will be required to formally establish the complete regulatory pathway (lines 354 to 355).

Minor comments:
If 1980/2309 make up 85+% of relative transcripts when c-di-GMP is induced, why is sporulation not as impacted between the WT strain and the Δ2 strain? Is this because of potential differences in expression of proteins between TY medium and sporulation agar or is there an alternative explanation?

We thank the reviewer for this thoughtful comment. We would first like to clarify that the ~85% combined relative abundance of CD1980.2 and CD2309 refers to the wild-type condition in TY medium, rather than to the elevated c-di-GMP condition.

To address the reviewer's question regarding growth conditions, we analyzed transcript abundance under sporulation conditions by RNA-seq of the wild-type strain grown for 16 h on 70:30 agar. CD1980.2 and CD2309 remained the two predominant paralogs, representing ~48% and ~43% of the total reads assigned to the five loci, respectively.

We also tested whether deletion of one or two major paralogs triggered compensatory upregulation of the remaining copies. qRT-PCR analysis did not reveal significant changes in expression of the remaining paralogs in the corresponding deletion mutants.

These results suggest that the relatively modest phenotype of the double mutant is unlikely to be explained either by condition-specific transcript redistribution or by compensatory transcriptional induction. Rather, low-abundance paralogs may be sufficient to support sporulation, or individual paralogs may contribute unequally to sporulation efficiency. These new data have now been incorporated into the revised Results section (lines 299 to 309) and are presented in the supplementary Fig. 8.

Fig 5A: Generally, C. difficile sporulation does not continue significantly beyond 24 h on 70:30 agar. The observation that sporulation frequency more than doubled between 24 h and 72 h may be artificially higher because of vegetative cell lysis over time. Are the total number of vegetative cells and spores counted at each time point similar or does this number decrease over time? It would be clarifying to provide, in the supplemental, phase contrast micrographs of each time point to show the continued increase in sporulation frequency versus accumulated cell lysis of non-sporulating vegetative cells at 48 h and 72 h versus 24 h.

We thank the reviewer for this insightful comment and agree that the apparent increase in sporulation frequency observed between 24 h and 72 h on 70:30 agar may be influenced, at least in part, by progressive lysis of vegetative cells over time, thereby artificially increasing the calculated proportion of spores. As this temporal increase is not central to the main conclusions of the study and does not affect the strong sporulation defect observed for the mutant strains, we have simplified the presentation of these data and removed the extended time-course analysis from the revised manuscript. We thank the reviewer for helping us improve the rigor of the analysis.

Fig. 6A: The statistical comparison should be between the Δ7 strain and the pPtet-dccA strain based on the results (lines 264-267), not between the WT and each mutant individually (could note as not significant, ns).

We thank the reviewer for this suggestion. We have revised the statistical analysis in Fig. 6A by performing multiple comparisons between all groups. This analysis now includes comparisons between the Δ7 strain and the pP$_{tet}$-*dccA* strain, as suggested, and is indicated as not significant (ns). In addition, we have applied the same revised statistical approach to Fig. 6B to ensure consistency across the dataset. The updated statistical results are shown in the revised figures.

Line 312 - “near perfect” consensus site for CD2386.1

We thank the reviewer for this comment. We have revised the text as suggested and added “near” to the description of the consensus site upstream of CD2386.1 in the manuscript (line 384).

Line 317-319 – Because of the suggestion that c-di-GMP may affect RgaR activity, did the authors check to see if transcription of any other known RgaR targets were impacted by the overexpression of DccA?

We thank the reviewer for this insightful suggestion. To address this point, we examined the expression of previously described RgaR-regulated genes upon DccA overexpression. All targets except spoZ displayed a significant but relatively modest decrease in expression upon DccA overexpression, with fold changes ranging from approximately 2.2- to 4.3-fold.

Overall, these results are consistent with the hypothesis that increased c-di-GMP levels may influence the expression of several RgaR-regulated genes. However, the moderate magnitude of these effects does not allow us to conclude whether this regulation occurs through a direct impact on RgaR activity or through indirect mechanisms. We have now included these

analyses in the revised Results section (lines 179 to 182) and expanded the Discussion accordingly (lines 388 to 394).

Reviewer #3 (Remarks to the Author):

Manuscript ID: NCOMMS-25-94626

Manuscript Title: A family of small proteins links c-di-GMP riboswitch signaling with sporulation in Clostridioides difficile

Author summary
The study by Lobo et al. identifies a conserved family of small, membrane-associated proteins that function as novel components linking c-di-GMP signaling to sporulation control in Clostridioides difficile. These genes are transcriptionally repressed by class I c-di-GMP–responsive riboswitches through premature termination. Overexpression of a single family member activates sporulation genes and enhances spore formation, whereas successive deletions of these genes lead to a stepwise decrease in sporulation efficiency, culminating in a severe defect when all family members are absent. Elevated intracellular c-di-GMP levels do not further inhibit sporulation in the complete deletion mutant, supporting the conclusion that c-di-GMP acts by repressing expression of these small-protein genes. Overall, this work establishes a mechanistic link between c-di-GMP signaling and developmental regulation, thereby broadening the known repertoire of bacterial second messenger outputs.

Review
This manuscript is well written and logically structured. The experimental design is sound, and the data presentation appears accurate and convincing. Overall, the study might be of broad interest to microbiologists interested in sec. messenger signaling, as it provides findings that connect c-di-GMP signaling to cellular differentiation processes in this case sporulation. This has been so far only reported for Streptomyces spec and Bacillus anthracis to my knowledge.
Nevertheless, several major points should be addressed to further strengthen the manuscript and permit publication, as outlined below.

General/ major comments
1. Terminology in c-di-GMP/second messenger signaling
Putative binding of c-di-GMP to newly identified proteins?
The terms c-di-GMP effector should be reserved for molecules that directly bind c-di-GMP, such as PilZ-domain proteins, transcription factors, degenerated GGDEF/EAL domain proteins, or c-di-GMP-responsive riboswitch RNAs. The proteins themselves, reported in this study, are likely to not directly bind c-di-GMP. However, this is in my view discriminated in the manuscript. Please provide reasonable arguments why this has not been tested in the current study (see below: bioinformatic/ structural analysis of “small-membrane proteins”).

Mechanistically, the riboswitch represents the true c-di-GMP effector, whereas the encoded membrane proteins function downstream as targets or outputs of the signaling pathway.
To ensure accurate and precise terminology throughout the manuscript, I suggest to revise sentences that may imply direct binding of c-di-GMP by these proteins. Phrasing such as the following would be more appropriate:

“X is a c-di-GMP–regulated protein whose expression is controlled by a c-di-GMP–responsive riboswitch,” or
“X functions in a c-di-GMP signaling output pathway that is transcriptionally regulated via a c-di-GMP–responsive riboswitch.”
In particular, corresponding statements in the abstract and introduction (e.g., lines 25, 36, 90, and 94) should be adjusted to convey this distinction clearly and consistently.

We thank the reviewer for this careful and insightful comment regarding terminology and conceptual clarity in c-di-GMP signaling. We fully agree that the term “c-di-GMP effector” should be restricted to molecules that directly bind c-di-GMP, such as PilZ-domain proteins, degenerate GGDEF/EAL proteins, transcription factors, or c-di-GMP-responsive riboswitches. In our study, the small membrane proteins described are not proposed to directly bind c-di-GMP, and this distinction was already intended in the manuscript, where their expression is linked to c-di-GMP via riboswitch-dependent transcriptional regulation.

We agree that direct binding of c-di-GMP to these proteins has not been tested. This was not the focus of the present study, which aimed to characterize the regulatory output of c-di-GMP signaling mediated through riboswitch-controlled gene expression rather than to identify new classes of direct c-di-GMP-binding proteins. Moreover, these proteins are exceptionally small (58 amino acids), lack any predicted conserved domains or recognizable c-di-GMP-binding motifs, and therefore provide no obvious bioinformatic indication that they would directly bind c-di-GMP. While we cannot formally exclude this possibility, it appears unlikely based on their size and predicted architecture. A dedicated biochemical and structural analysis would nevertheless be required to address this question and is beyond the scope of the current work.

In response to the reviewer’s suggestion, we have carefully revised the manuscript to avoid any terminology that could imply direct binding of c-di-GMP by these proteins. We now consistently refer to them as c-di-GMP-regulated proteins whose expression is controlled via c-di-GMP–responsive riboswitches, and we have modified the Abstract and Introduction accordingly to ensure conceptual clarity and consistency throughout the manuscript.

2. Relation to previous transcriptional analyses (e.g., McKee et al., mSphere 2018)
The authors should clarify how their RNA-seq based approach differs from previous transcriptomic studies, such as that of McKee et al. (2018). Specifically, were genes CD1424, CD1990, CD2309, and CD3368 also identified in earlier datasets as strongly repressed under conditions of elevated c-di-GMP? It is not entirely clear whether the present transcriptomic analysis newly identified these small membrane-protein genes or confirmed prior observations with greater resolution. Providing a direct comparison to the earlier studies would help position this work within the existing literature and underscore the novelty of the findings, potentially.

We thank the reviewer for this insightful comment and agree that the relationship between our study and previous transcriptomic analyses should be clarified.

Previous work by McKee et al. (mSphere, 2018) also investigated the consequences of elevated intracellular c-di-GMP levels, using plasmid-based expression of *dccA* under a nisin-

inducible promoter, whereas in the present study *dccA* was induced from a chromosomally integrated *Ptet-dccA* construct. In both studies, several class I riboswitch-associated loci were found to be negatively regulated under high c-di-GMP conditions. However, the biological interpretation of these loci differs substantially. In McKee et al., three downstream genes were annotated only as hypothetical proteins (CD1990.3, CD2309, and CD3368.2), without recognition that they encode unusually small proteins (~58 aa), and no connection was made between these genes. In addition, no coding sequence was assigned downstream of Cdi1_6 and Cdi1_10, leading the authors to suggest that these riboswitches might be nonfunctional or act independently of downstream gene regulation.

By contrast, our analyses show that all five riboswitches are functional and control genes encoding highly similar 58-aa membrane-associated proteins that constitute a conserved multicopy family. We further demonstrate that these proteins collectively promote sporulation. Thus, while our transcriptomic observations are broadly consistent with previous datasets, the present study provides a fundamentally new interpretation of these loci by revealing their shared identity, conservation, and physiological function. We have now clarified this point in the revised manuscript (lines 149 to 154).

3. Schematic overview of c-di-GMP signaling
I recommend to include a schematic overview illustrating c-di-GMP metabolism especially its regulatory outputs in Clostridioides difficile. This figure would effectively contextualize the newly identified c-di-GMP-responsive genes encoding small membrane proteins within the broader signaling network. Such a diagram could include additionally key known functions of c-di-GMP in C. difficile: riboswitch-mediated control of motility (flgB operon via class I riboswitches), toxin production, surface adhesion and type IV pilus assembly (pilA1 via class II riboswitch).

We thank the reviewer for this helpful suggestion. To better place our findings in the broader context of c-di-GMP signaling in *C. difficile*, we have added a new schematic overview (new Fig. 7) at the end of the Results section (lines 335 to 338). This figure summarizes c-di-GMP metabolism and its major known regulatory outputs in *C. difficile*, including riboswitch-mediated control of motility (*flgB* operon), type IV pilus assembly (*pilA1*), toxin production, and surface-associated behaviors, while highlighting the newly identified small membrane proteins described in this study as an additional c-di-GMP-regulated pathway linked to sporulation.

4. Bioinformatic/ structural analysis of small membrane proteins
In my view, the authors should complement their AA sequence alignments with secondary structure predictions and AlphaFold models, including superimpositions of homologs (maybe also from related species?). These analyses would provide readers with a clear visual representation of the proteins' predicted structures and conserved features. They could be presented as new panels in an existing figure (e.g., Figure 3) or as a supplementary figure.
I predicted the protein structure of CD2309 with alpha-fold, obviously it does predeict (more or less with high confidence) 3-4 a-helices arranged in a parallel manner. Please provide more information on the putative hydrophobic helix at the C-Terminus! Is the C-Terminus indeed

critical for membrane insertion. I would suggest localization assays with truncated versions based on alpha-fold predictions!

Suggestion for an additional paragraph within the manuscript:
The small membrane proteins encoded by CD1424, CD1990, CD2309, and CD3368 in Clostridioides difficile strain 630 exhibit high sequence conservation, particularly in their predicted helical regions, as revealed by multiple sequence alignments. Secondary structure predictions using PSIPRED confirm 3-4 transmembrane alpha-helices in each protein, with the C-terminal helix displaying strong hydrophobicity suggestive of membrane anchoring. AlphaFold2 models for CD2309 demonstrate high-confidence predictions (pLDDT > 90 for core helices) of four parallel alpha-helices forming a compact bundle, consistent with the reviewer's observation and alignments with homologs from related Clostridia species such as Clostridium sporogenes.

We thank the reviewer for this thoughtful and constructive suggestion. To further characterize this protein family, we performed AlphaFold3-based structural analyses of the five riboswitch-associated paralogs (CD2309, CD1424, CD1980.2, CD1990.3, and CD3368.2). These analyses confirmed a highly conserved global fold composed predominantly of α-helices, with strong structural similarity across paralogs.

Importantly, all models displayed a conserved C-terminal helix with a pronounced hydrophobic surface patch, consistent with a role in membrane association or membrane interaction. These *in silico* observations are in agreement with our biochemical fractionation experiments showing membrane enrichment of CD1980.2 and CD2309. In addition, complementary electrostatic and hydrophobicity analyses revealed conserved surface features, including a hydrophobic C-terminal region and a charged helical core, further supporting a membrane-associated architecture potentially involved in molecular interactions. We have now incorporated these analyses into a new supplementary figure (Supplementary Figure 4) and added the corresponding text to the revised manuscript (lines 185 to 199).

We note that structural predictions alone do not allow definitive conclusions regarding topology or oligomeric state. We therefore favour a cautious interpretation. Regarding the reviewer's suggestion of localization assays using truncated variants, we agree that this would be an informative approach to directly test the role of the C-terminal helix. However, these experiments would require substantial additional strain construction and validation and are beyond the scope of the present study. We now mention this as an important direction for future work (lines 411 to 413).

Finally, we re-examined homolog distribution using BLAST and structural database searches. These analyses did not identify convincing homologs outside the *Clostridioides difficile* species complex (including cryptic lineages) as previously mentioned in line 147, and we did not detect clear counterparts in *Clostridium sporogenes* or other *Clostridium* species.

5. Relevance of small membrane proteins during spore formation/ spore germination?
Small membrane proteins like those encoded by CD1424, CD1990, CD2309, and CD3368 show potential relevance to spore formation and germination due to their predicted transmembrane helical structures, which could facilitate signaling or structural roles at the

membrane during sporulation stages. While direct localization via fluorescence microscopy poses challenges due to their small size and hydrophobicity, transcriptional analysis such as qRT-PCR during sporulation time courses (e.g., monitoring expression peaks at stages II-IV) offers a feasible, indirect way to link them to sporulation or germination processes.

We thank the reviewer for this insightful suggestion regarding the potential role of these small membrane proteins during sporulation and germination.

We agree that their predicted membrane-associated helices are consistent with a possible function at the membrane during developmental processes. In this study, we already addressed the regulation and functional relevance of this protein family in the context of sporulation using complementary approaches, including RNA-seq analyses under high c-di-GMP conditions and during growth on sporulation agar, as well as functional studies showing that deletion of these genes severely impairs sporulation, whereas overexpression of *CD1980.2* promotes spore formation. Together, these data support a role for this protein family in promoting sporulation rather than germination, although the precise stage(s) at which they act remain to be determined.

To further address the reviewer's suggestion, we performed qRT-PCR analyses of the representative small-protein gene *CD2309* together with additional c-di-GMP riboswitch-controlled genes (*CD2831*, *pilA1*, and *ppep-1*) in the wild-type strain grown under sporulation conditions (70:30 medium). To independently evaluate the effects of growth stage and growth condition, gene expression was compared between early (4 h) and late (16 h) time points in liquid medium, and between liquid and solid (70:30 agar) cultures at 16 h. Under all conditions tested, we did not observe significant changes in expression for any of these genes. These results indicate that expression of these genes is not strongly regulated during sporulation and suggest that their role is likely permissive rather than stage-specific. Importantly, interpretation of population-level transcriptional analyses in *C. difficile* is limited by the highly asynchronous nature of sporulation, with stages II–IV coexisting within the same culture, which reduces temporal resolution. Together with the absence of major transcriptional changes, this suggests that these proteins may act through constitutive or basal expression, and that, consistent with current views (PMID: 33640237), any associated c-di-GMP dynamics are likely subtle and/or spatially localized rather than driven by large global changes. These new data have now been incorporated into the revised Results section (lines lines 278–284; Supplementary Fig. 6B-C) and their implications discussed in the Discussion (lines 358 to 368).

Because population-level transcriptional analyses provide only limited temporal resolution in this system, resolving the precise timing and localization of these proteins during sporulation will require future single-cell or biochemical approaches, which are beyond the scope of the present study.

In view of the substantial concerns detailed above, I regret to conclude that in my view the manuscript is most likely not suitable for publication in its present form and therefore must recommend major revision!
Kind regards

We thank the reviewer for carefully evaluating our manuscript and for the detailed and constructive comments provided throughout the review process. We have carefully addressed all concerns raised, including additional analyses (structural predictions and sequence conservation), clarification of terminology regarding c-di-GMP signaling, refinement of the manuscript text, and improved interpretation of previous transcriptomic datasets.

We believe that these extensive revisions have significantly strengthened the manuscript and improved its clarity, rigor, and overall contribution to the field. We respectfully hope that the revised version will now meet the standards for publication.
