# Supplementary material for: A family of small proteins links c-di-GMP riboswitch signaling with sporulation in Clostridioides difficile
Source: Nat Commun. 2026 Oct 4;17:11319. doi: 10.1038/s41467-026-78452-6 (PMC13724677; doi:10.1038/s41467-026-78452-6)
Supplement: Supplementary file 3 [file 41467_2026_78452_MOESM1_ESM.pdf]

A family of small proteins links c-di-GMP riboswitch signaling with sporulation in *Clostridioides difficile*

Adriana Badilla Lobo,[a] Anne-Judith Waligora-Dupriet,[b], Louise Penven,[a] Roland Seifert,[c] César Rodríguez,[d] Frédéric Barbut,[b,e] Olga Soutourina[a] and Johann Peltier[a]*

[a]Université Paris-Saclay, CEA, CNRS, Institute for Integrative Biology of the Cell (I2BC), Gif-sur-Yvette, France.
[b]Université Paris Cité, INSERM, UMRS 1139, FPRM, Paris, France.
[c]Institute of Pharmacology & Research Core Unit Metabolomics, Hannover Medical School, Hannover, Germany.
[d]Facultad de Microbiología & Centro de Investigación en Enfermedades Tropicales, Universidad de Costa Rica, San José, Costa Rica
[e] National Reference Laboratory for *Clostridioides difficile*, Assistance Publique-Hôpitaux de Paris Hôpital Saint-Antoine, France.

Supplementary information

Supplementary Figure 1. RNA-seq visualization of the *dccA–CD1419* locus.

Supplementary Figure 2. A remnant ORF associated with the c-di-GMP-responding riboswitch Cdi1_7 encodes a truncated peptide homologous to CD1980.2.

Supplementary Figure 3. RgaR regulates three CD1980.2 orthologs not linked to a c-di-GMP riboswitch.

Supplementary Figure 4. Structural characterization of the riboswitch-associated small protein family.

Supplementary Figure 5. C-di-GMP requires the riboswitches to modulate the expression of the downstream genes.

Supplementary Figure 6. Sporulation-promoting activity of CD1980.2 in UK1 and stable expression of c-di-GMP riboswitch-associated genes under sporulation conditions.

Supplementary Figure 7. PCR verification of the seven deletions in the in *C. difficile* 630Δ*erm* Δ7 mutant.

Supplementary Figure 8. Low-abundance small protein paralogs contribute to sporulation in *Clostridioides difficile*.

Supplementary Figure 9. AlphaFold confidence assessment for CD1424.

Supplementary Figure 10. AlphaFold confidence assessment for CD1980.2.

Supplementary Figure 11. AlphaFold confidence assessment for CD1990.3.

Supplementary Figure 12. AlphaFold confidence assessment for CD2309.

Supplementary Figure 13. AlphaFold confidence assessment for CD3368.2.

Supplementary Figure 14. AlphaFold confidence assessment for CD2309 homodimer.

Supplementary Table 1. Strains and plasmids used in this study.

Supplementary Table 2. Oligonucleotides used in this study.

Supplementary Table 3. AlphaFold model quality metrics for protein structures.

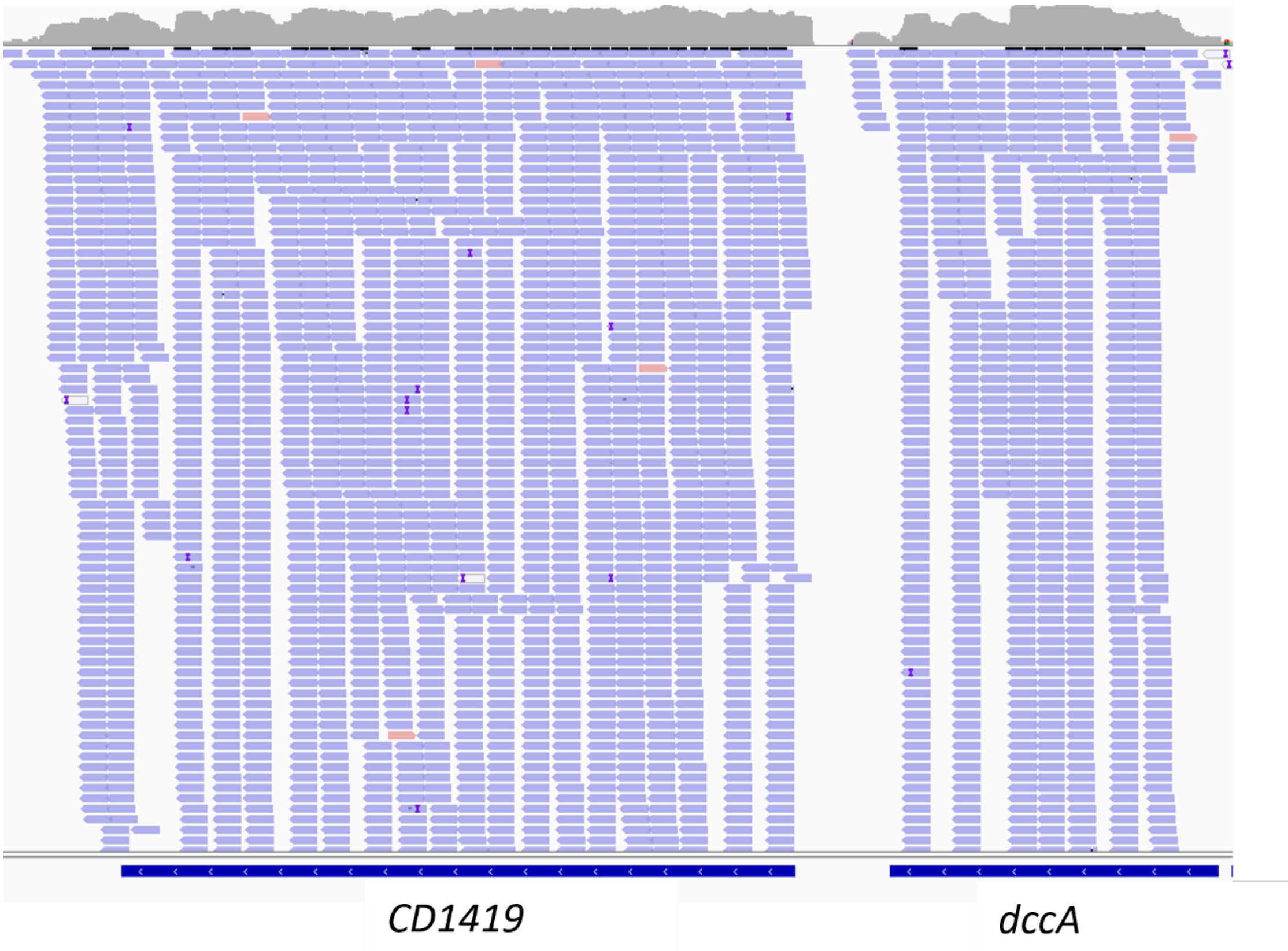

Supplementary Figure 1: RNA-seq visualization of the *dccA–CD1419* locus. RNA-seq reads from the P_{tet}-*dccA* strain were visualized using IGV. A representative genomic region spanning *dccA–CD1419* is shown. Reads aligned to the "+" strand are displayed in red and reads aligned to the "–" strand in blue.

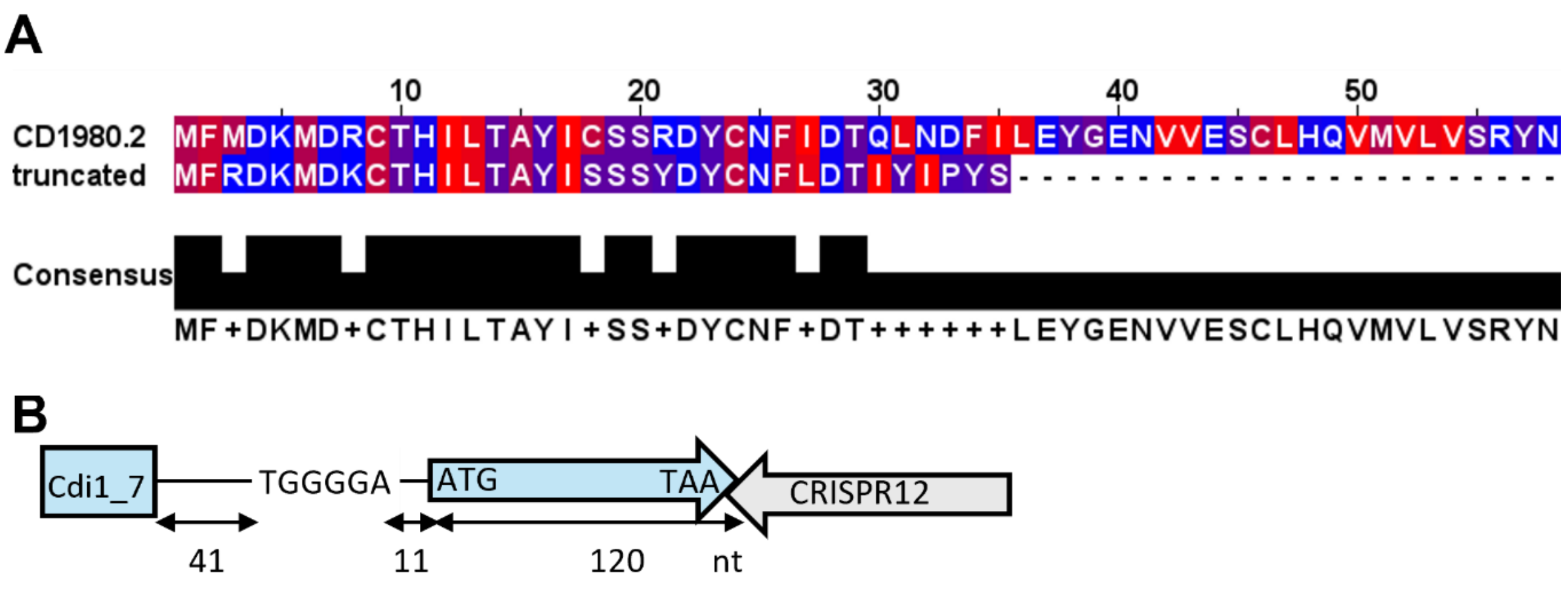

Supplementary Figure 2. A remnant ORF associated with the c-di-GMP-responding riboswitch Cdi1_7 encodes a truncated peptide homologous to CD1980.2. (A) Alignment of the small protein CD1980.2 with a truncated protein encoded by a remnant ORF associated with Cdi1_7. The hydrophobic and positively charged amino acids are indicated in red and blue, respectively. **(B)** Schematic representation of the Cdi1_7 riboswitch locus. The downstream remnant CDS is depicted as an arrow with the start (ATG) and the stop (TAA) codons, and the size of the CDS is indicated. The CRISPR array 12 located directly downstream disrupts the CDS. The putative RBS sequence (TGGGGA) is represented and the number of nucleotides between the RBS and the riboswitch and between the RBS and the CDS are indicated.

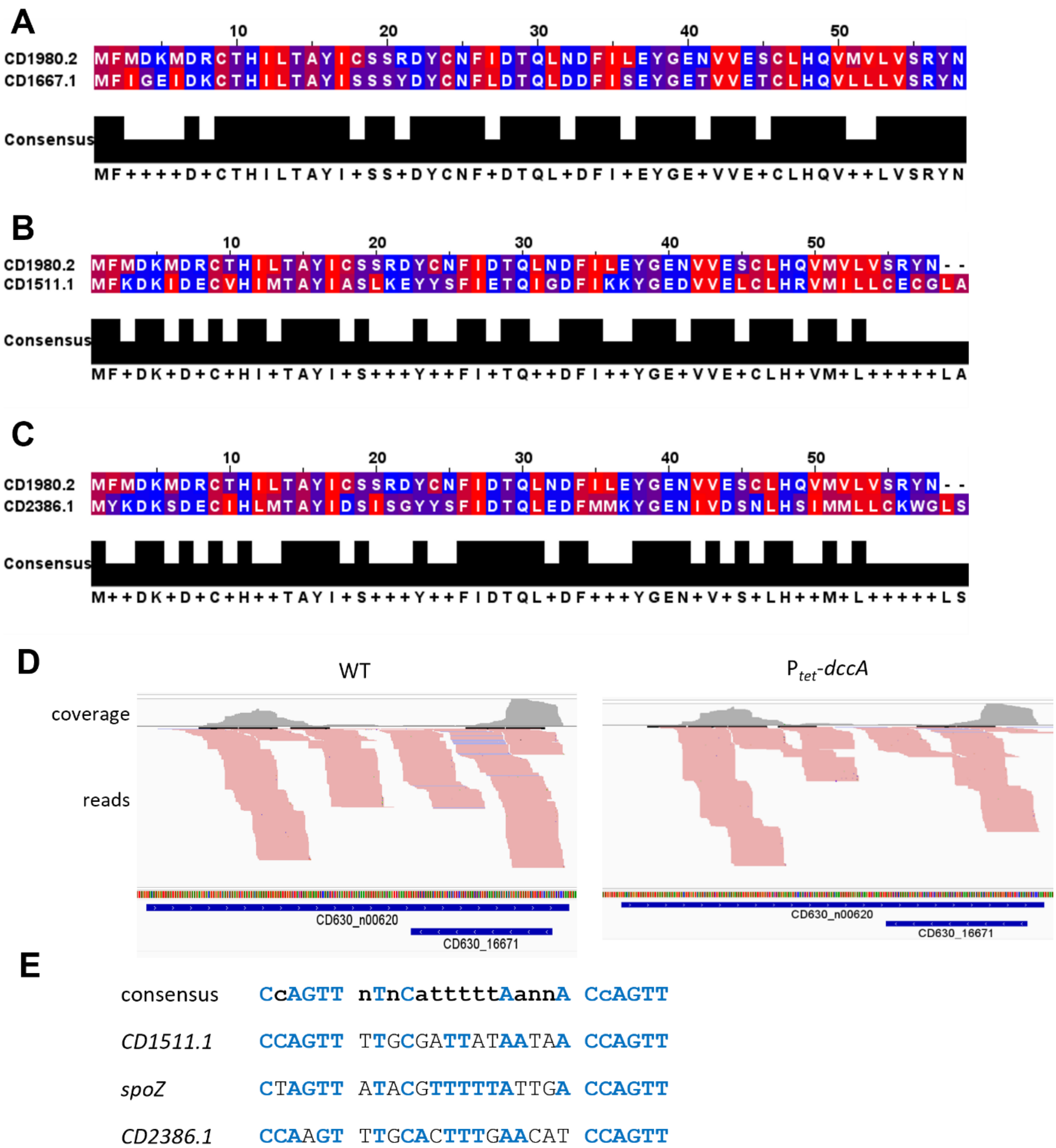

Supplementary Figure 3. RgaR regulates three CD1980.2 orthologs not linked to a c-di-GMP riboswitch. (A to C) Alignment of the small protein CD1980.2 with the small protein CD1667.1 **(A)**, CD1511.1 **(B)** or CD2386.1 **(C)**. The hydrophobic and positively charged amino acids are indicated in red and blue, respectively. **(D)** Visualization of RNA-seq data of the *CD1667.1*/*spoZ* (CD630_n00620) region from C. *difficile* wild type (WT) and P_{tet}-*dccA* , in which the diguanylate cyclase-encoding gene *dccA* is expressed from a chromosomally integrated anhydrotetracycline (ATc)-inducible promoter. Cultures for RNA-seq were grown in TY with 250 ng/ml ATc. "+" strand reads are shown in red, and "-" strand reads are shown in blue. Data are representative of four experiments. **(E)** Alignment of the putative RgaR binding sites with the promoters of *CD1511.1*, *spoZ* and *CD2386.1*.

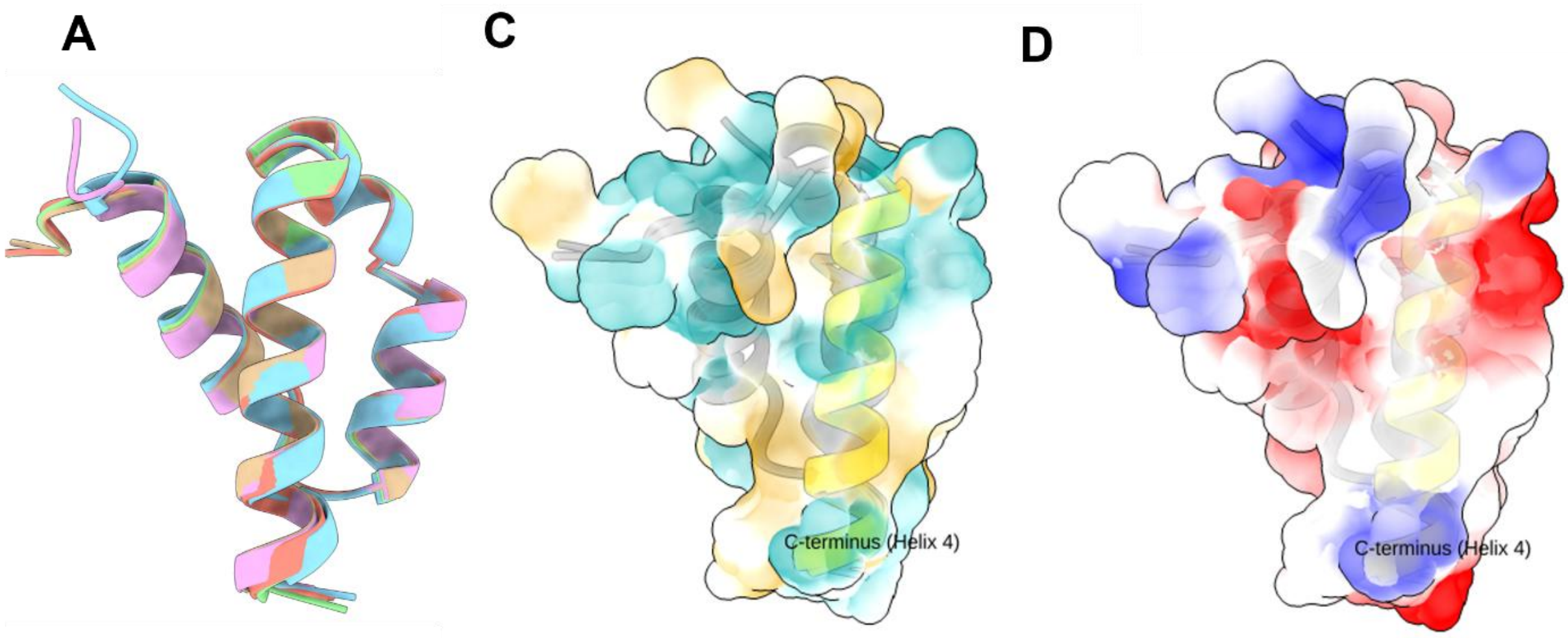

B

Protein Model	H1 / H2 Angle (Oblique)	H1 / H3 Angle (Antiparallel)	H1 / H4 Angle (Anchor)	Anchor RMSD (Å)
Model 1 (CD2309)	107.0°	155.2°	49.9°	0.000 (Ref)
Model 2 (CD1424)	113.3°	143.4°	37.4°	0.192
Model 3 (CD1980.2)	96.8°	159.3°	61.6°	0.223
Model 4 (CD1990.3)	94.0°	152.9°	63.9°	0.216
Model 5 (CD3368.2)	106.1°	155.1°	49.9°	0.215

Supplementary Figure 4. Structural characterization of the riboswitch-associated small protein family. (A) Structural superposition of AlphaFold3-predicted models of the five paralogs (CD2309, CD1424, CD1980_2, CD1990_3, and CD3368_2 demonstrating a conserved global architecture (global RMSD < 0.5 Å). Protein backbones are shown as cartoon representations, illustrating the strong structural similarity across paralogs despite moderate sequence divergence. (**B**) Quantitative interhelical angles and C-terminal anchor RMSD values for the five superposed models, derived from vector-based helical axis analysis. Helices 1 and 3 adopt an antiparallel arrangement (143.4–159.3°); Helices 2 and 4 adopt oblique orientations. Anchor RMSD values (0.19–0.22 Å) confirm exceptional geometric conservation of Helix 4 across all paralogs. (**C**) Molecular lipophilicity potential (MLP) mapped onto the solvent-excluded surfaces of the five superposed models. Yellow, hydrophobic; cyan, hydrophilic. The C-terminal Helix 4 (residues 40–57), rendered as a yellow cartoon, consistently exhibits a pronounced outward-facing hydrophobic surface patch across all models, supporting its proposed role as a membrane anchor. (**D**) Electrostatic surface potential of the four-helix bundle reveals a putative interaction interface. The solvent-excluded surface of the representative model (CD2309) is colored according to Coulombic electrostatic potential (blue, positive; red, negative; white, neutral). A prominent electropositive patch is located on the exposed surface of the helical core (Helices 1–3), suggesting a potential binding interface for negatively charged ligands or protein partners. In contrast, the C-terminal helix (Helix 4) is largely electrostatically neutral, consistent with its lipophilic character and role in membrane association. Molecular graphics were generated using UCSF ChimeraX.

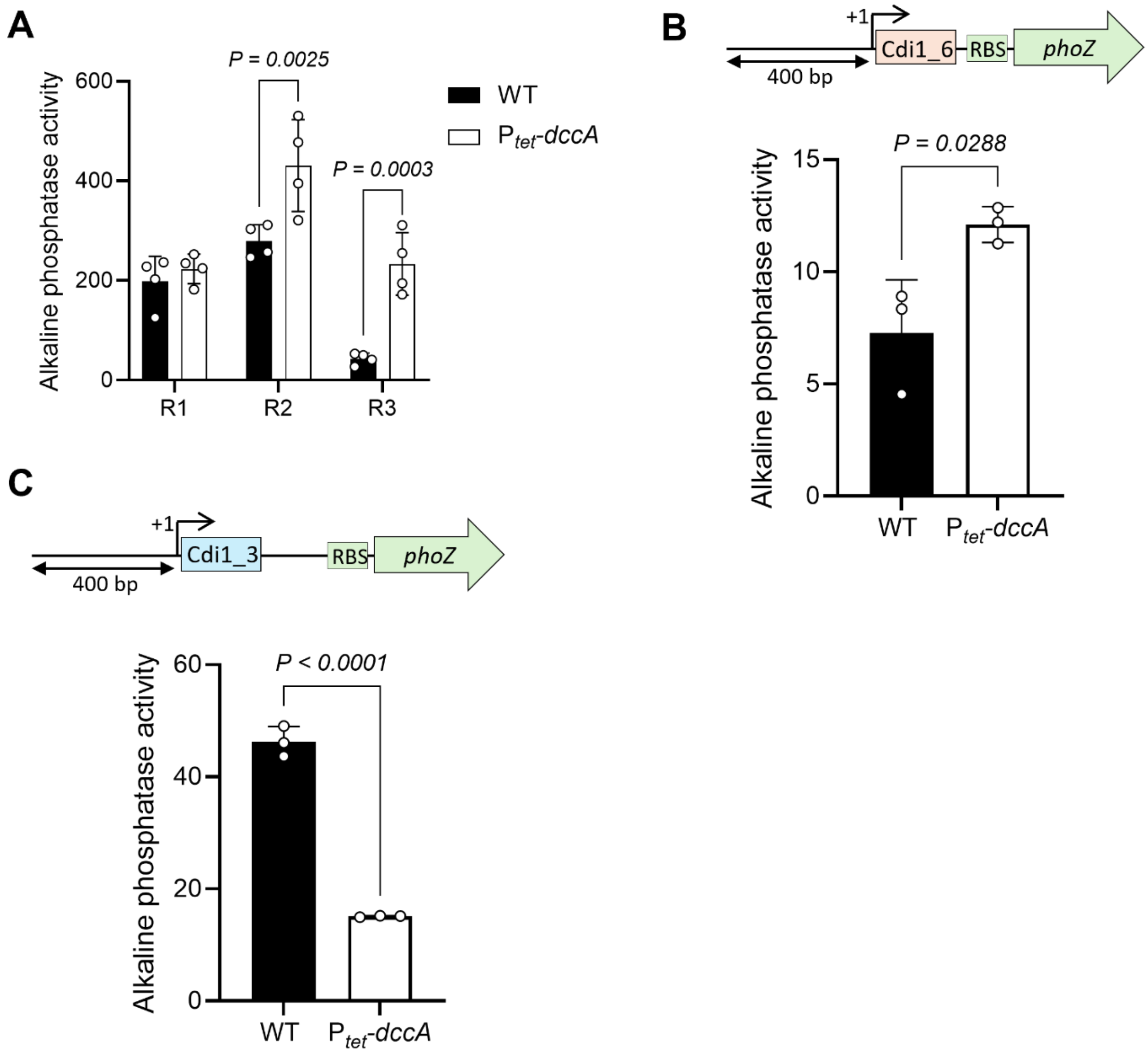

Supplementary Figure 5. C-di-GMP requires the riboswitches to modulate the expression of the downstream genes. (A) Alkaline phosphatase activity of the Cdi1_9::*phoZ* reporter fusions of various lengths (as depicted in Fig. 4A) in the wild-type (WT) and the P_{tet}-*dccA* strains, in which the diguanylate cyclase-encoding gene *dccA* is expressed from a chromosomally integrated anhydrotetracycline (ATc)-inducible promoter. Strains were grown in the exponential phase in TY with 25 ng/ml ATc. Bars represent means ± SD (*n* = 4 independent experiments). Statistical significance was assessed by a two-way ANOVA followed by a Sidak's multiple comparisons test. **(B)** Alkaline phosphatase activity of the Cdi1_6::*phoZ* reporter fusion in the WT and the P_{tet}-*dccA* strains. Strains were grown in the exponential phase in TY with 250 ng/ml ATc. A schematic of the Cdi1_6::*phoZ* transcriptional fusion carried on a plasmid is shown. Bars represent means ± SD (*n* = 3 independent experiments). Statistical significance was assessed by an unpaired two-sided Student's *t* test. **(C)** Alkaline phosphatase activity of the Cdi1_3::*phoZ* reporter fusion in the WT and the P_{tet}-*dccA* strains. Strains were grown in the exponential phase in TY with 250 ng/ml ATc. A schematic of the Cdi1_3::*phoZ* transcriptional fusion carried on a plasmid is shown. Bars represent means ± SD (*n* = 3 independent experiments). Statistical significance was assessed by an unpaired two-sided Student's *t* test. Source data are provided as a Source Data file.

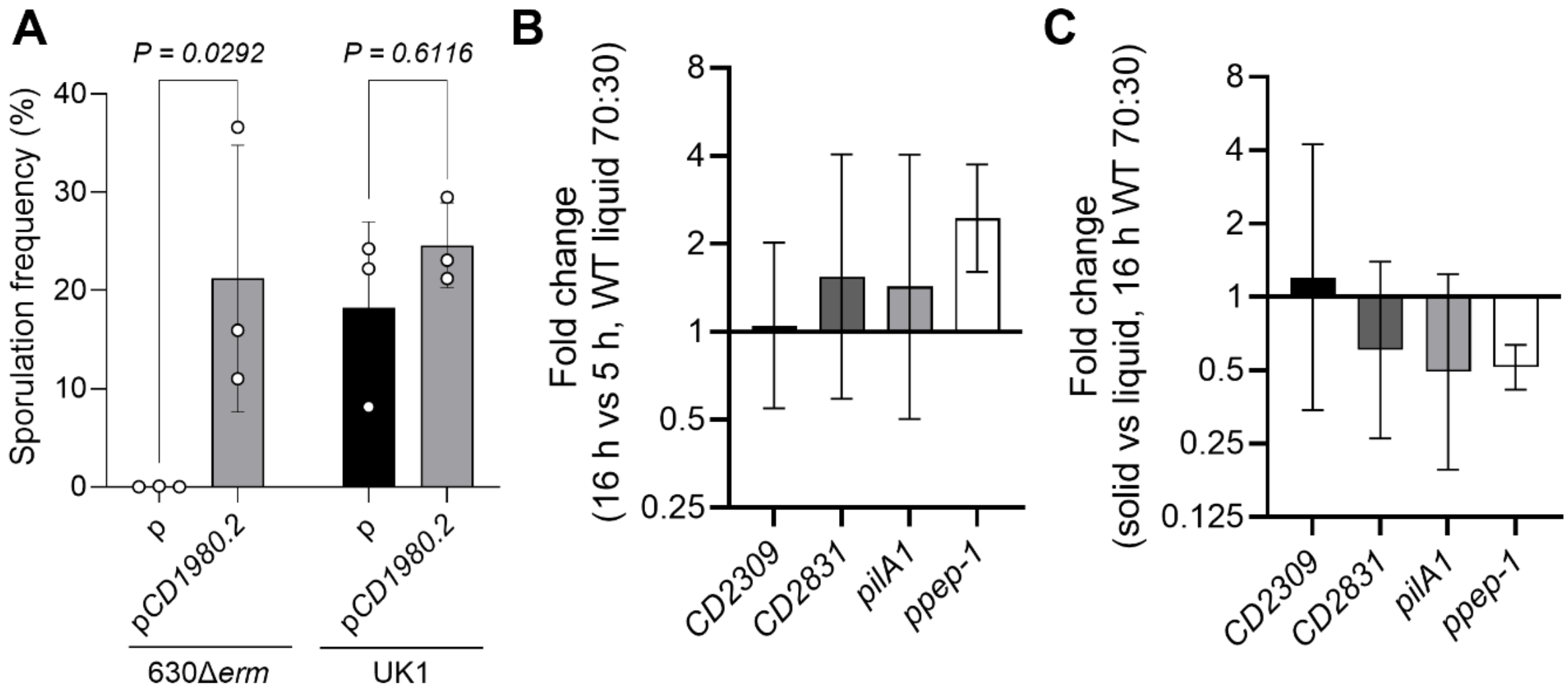

Supplementary Figure 6. Sporulation-promoting activity of CD1980.2 in UK1 and stable expression of c-di-GMP riboswitch-associated genes under sporulation conditions. (A) Overexpression of *CD1980.2* promotes sporulation in 630Δ*erm* but not in UK1 in liquid 70:30 medium. Ethanol-resistant spore formation of *C. difficile* 630Δ*erm*, and UK1 carrying an empty plasmid (p) or a plasmid expressing Cdi1_6-*CD1980.2* under the control of the P_{tet} promoter (p*CD1980.2*) grown for 24h in liquid 70:30 medium in the presence of 250 ng/mL ATc. Bars represent means ± SD (n = 3 independent experiments). Statistical significance was assessed by a two-way ANOVA followed by a Sidak's multiple comparison test. **(B)** and **(C)** Expression analysis of representative c-di-GMP riboswitch-associated genes during growth under sporulation conditions. Transcript levels of *CD2309, CD2831*, *pilA1* and *ppep-1* were measured by qRT-PCR in the wild-type strain grown in 70:30 medium. **(B)** Comparison of transcript levels between 4 h and 16 h in liquid culture. **(C)** Comparison of transcript levels between liquid and solid (70:30 agar) cultures after 16 h of growth. Bars represent means ± SD (n = 3 independent experiments). Source data are provided as a Source Data file.

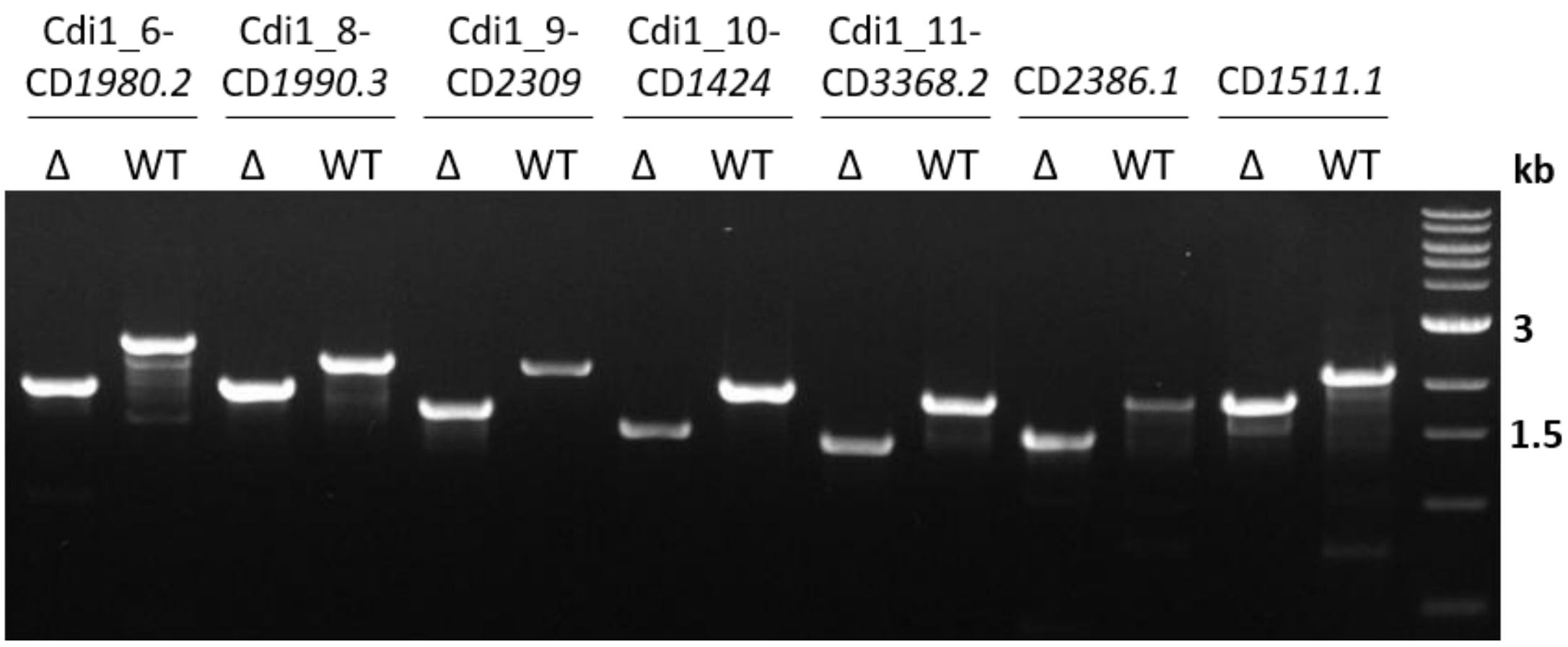

Supplementary Figure 7. PCR verification of the seven deletions in the *C. difficile* 630Δ*erm* Δ7 mutant. PCR amplification from wild type (WT) and Δ7 (Δ) to verify the indicated alleles. Source data are provided as a Source Data file.

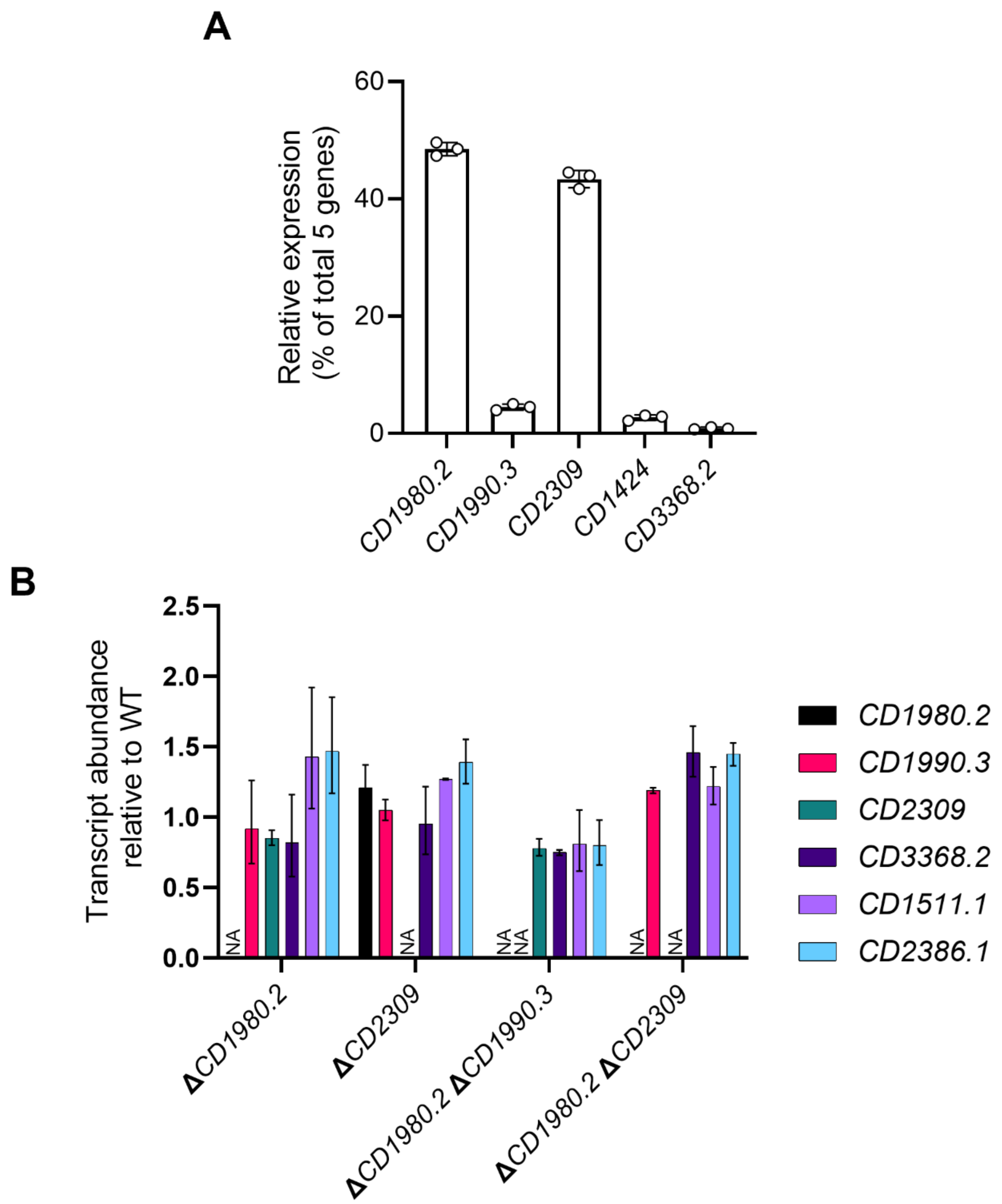

Supplementary Figure 8. Low-abundance small protein paralogs contribute to sporulation in *Clostridioides difficile*. (A) Relative mRNA abundance of the five small protein-encoding genes estimated from RNA-seq data obtained from the WT strain grown for 16 h on 70:30 sporulation agar supplemented with 250 ng/mL ATc. Bars represent means ± SD (n = 3 independent experiments). **(B)** qRT-PCR analysis showing expression levels of selected paralogs in 630Δ*erm* wild type and indicated deletion mutants grown in the exponential phase in TY medium. Bars represent means ± SD (n = 2 independent experiments). NA, not applicable, indicating absence of amplification for the deleted target gene in the corresponding mutant strain (Ct > 30).

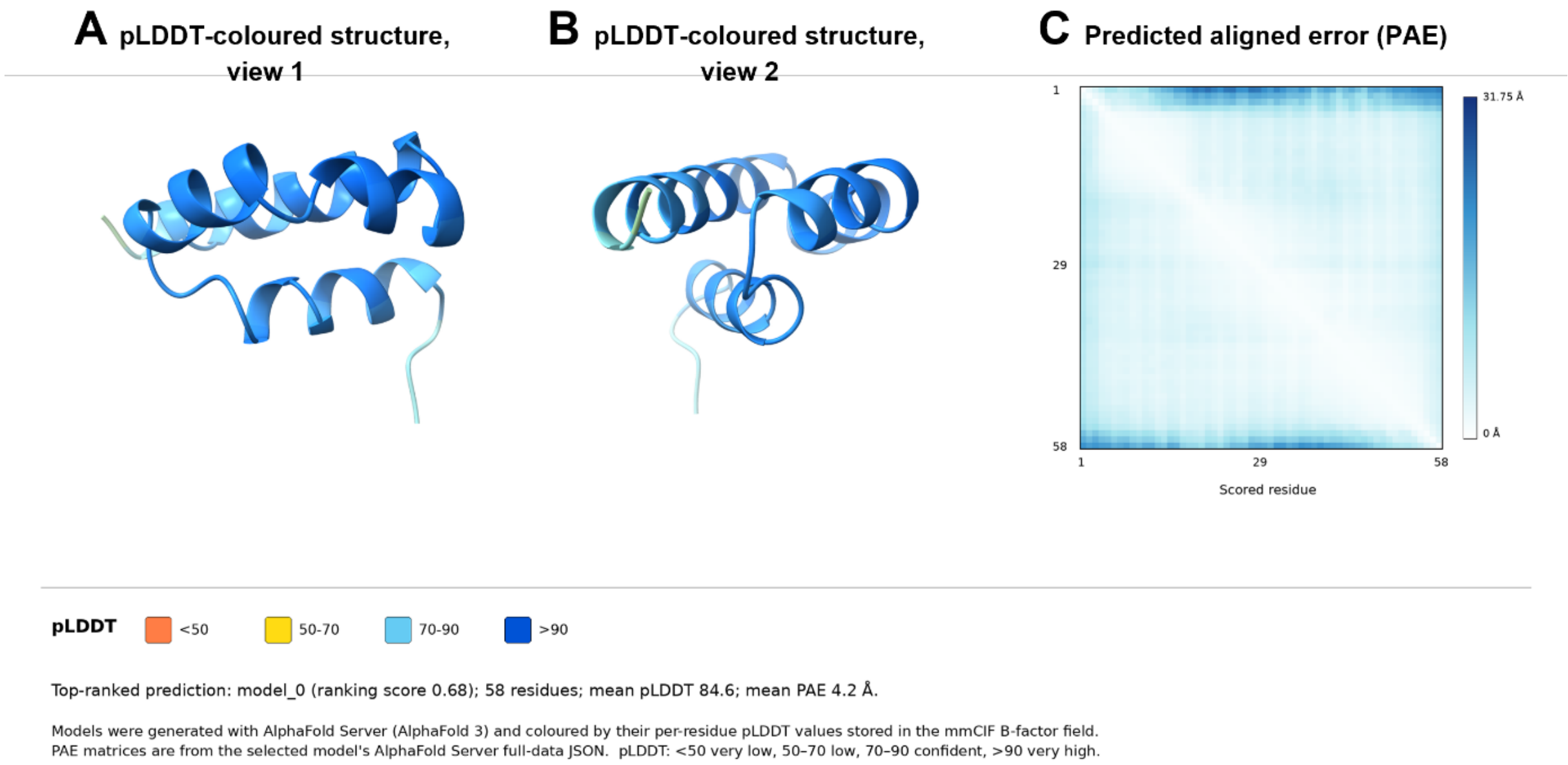

Supplementary Figure 9. AlphaFold confidence assessment for CD1424. (A, B) Two orthogonal cartoon views of the top-ranked AlphaFold 3 prediction, coloured by pLDDT: dark blue, >90; light blue, 70–90; yellow, 50–70; orange, <50. **(C)** PAE matrix for the same prediction; lower values indicate greater confidence in the relative placement of residue pairs. The black divider in the homodimer PAE plot delineates chains A and B. The selected candidate index, ranking score, mean pLDDT and mean PAE are listed in Supplementary Table S1.

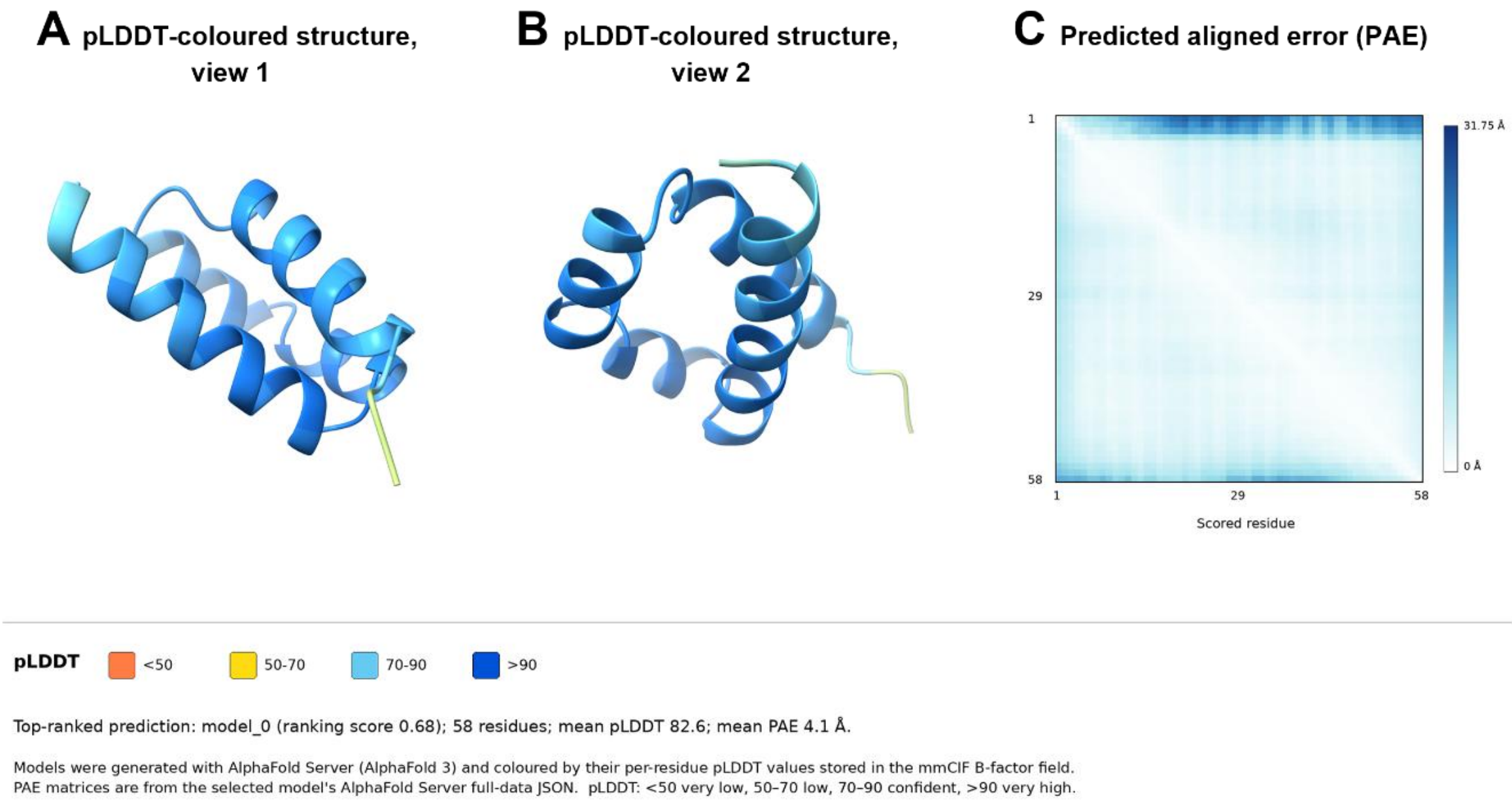

Supplementary Figure 10. AlphaFold confidence assessment for CD1980.2. (A, B) Two orthogonal cartoon views of the top-ranked AlphaFold 3 prediction, coloured by pLDDT: dark blue, >90; light blue, 70–90; yellow, 50–70; orange, <50. **(C)** PAE matrix for the same prediction; lower values indicate greater confidence in the relative placement of residue pairs. The black divider in the homodimer PAE plot delineates chains A and B. The selected candidate index, ranking score, mean pLDDT and mean PAE are listed in Supplementary Table S1.

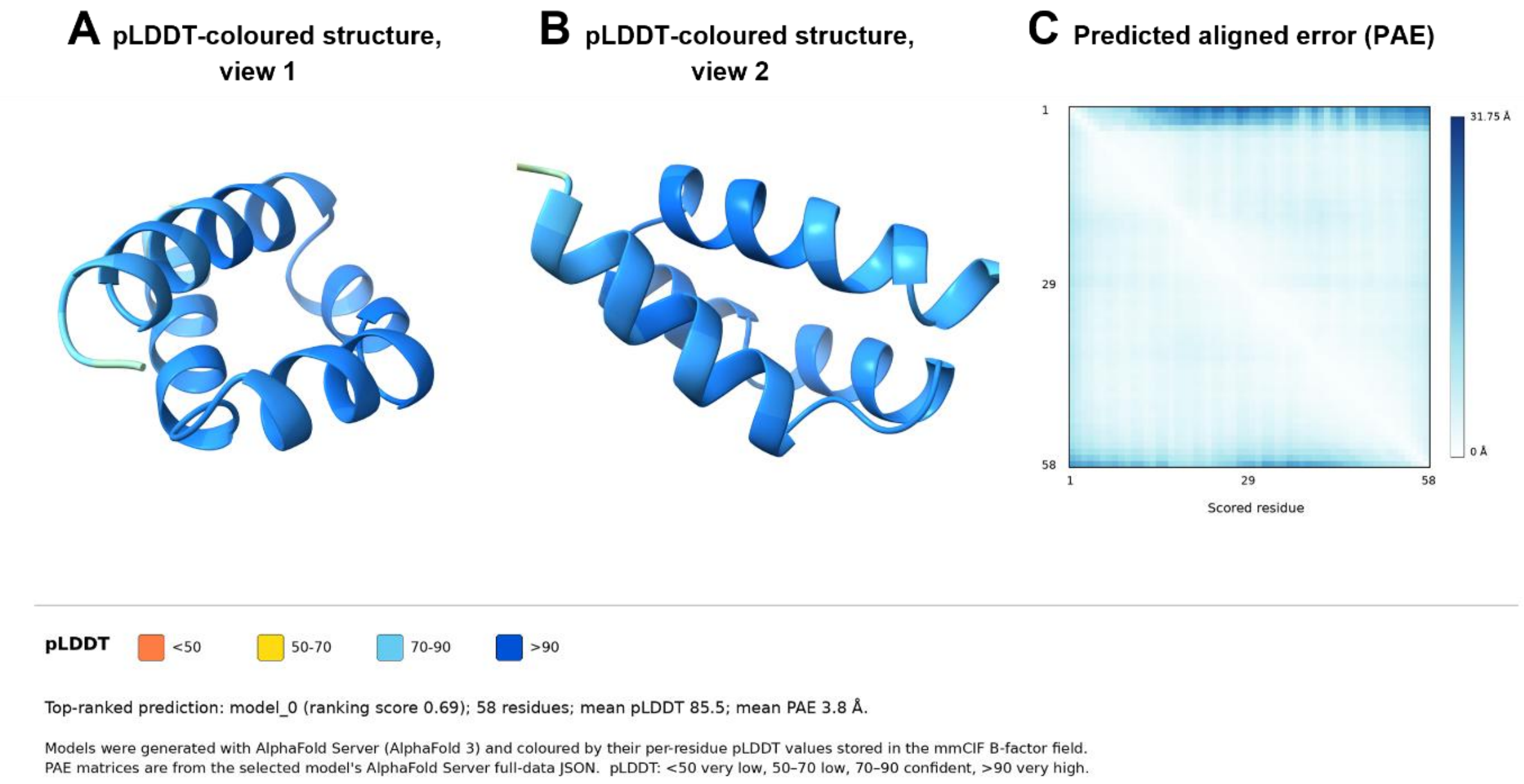

Supplementary Figure 11. AlphaFold confidence assessment for CD1990.3. (A, B) Two orthogonal cartoon views of the top-ranked AlphaFold 3 prediction, coloured by pLDDT: dark blue, >90; light blue, 70–90; yellow, 50–70; orange, <50. **(C)** PAE matrix for the same prediction; lower values indicate greater confidence in the relative placement of residue pairs. The black divider in the homodimer PAE plot delineates chains A and B. The selected candidate index, ranking score, mean pLDDT and mean PAE are listed in Supplementary Table S1.

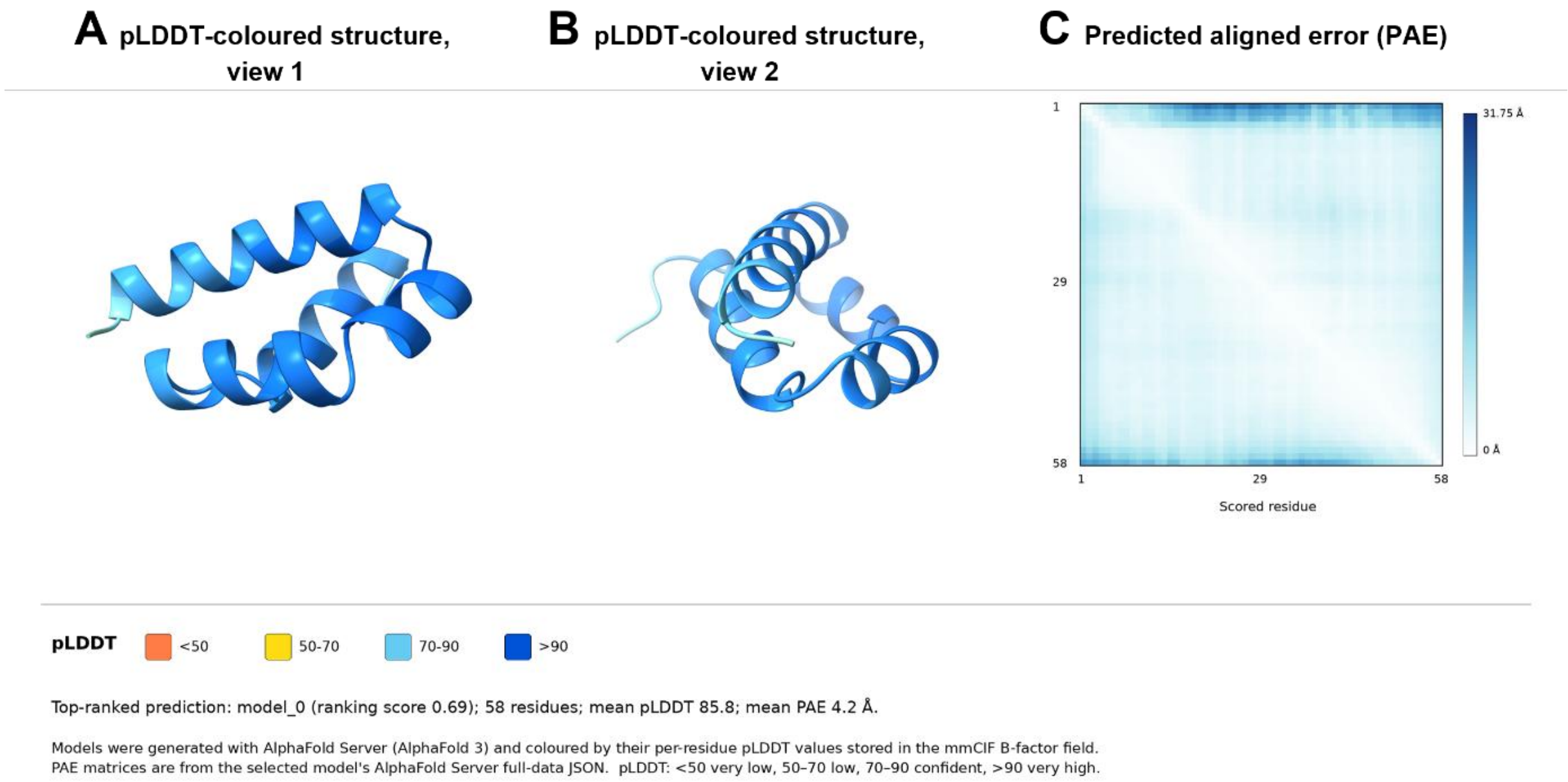

Supplementary Figure 12. AlphaFold confidence assessment for CD2309. (A, B) Two orthogonal cartoon views of the top-ranked AlphaFold 3 prediction, coloured by pLDDT: dark blue, >90; light blue, 70–90; yellow, 50–70; orange, <50. **(C)** PAE matrix for the same prediction; lower values indicate greater confidence in the relative placement of residue pairs. The black divider in the homodimer PAE plot delineates chains A and B. The selected candidate index, ranking score, mean pLDDT and mean PAE are listed in Supplementary Table S1.

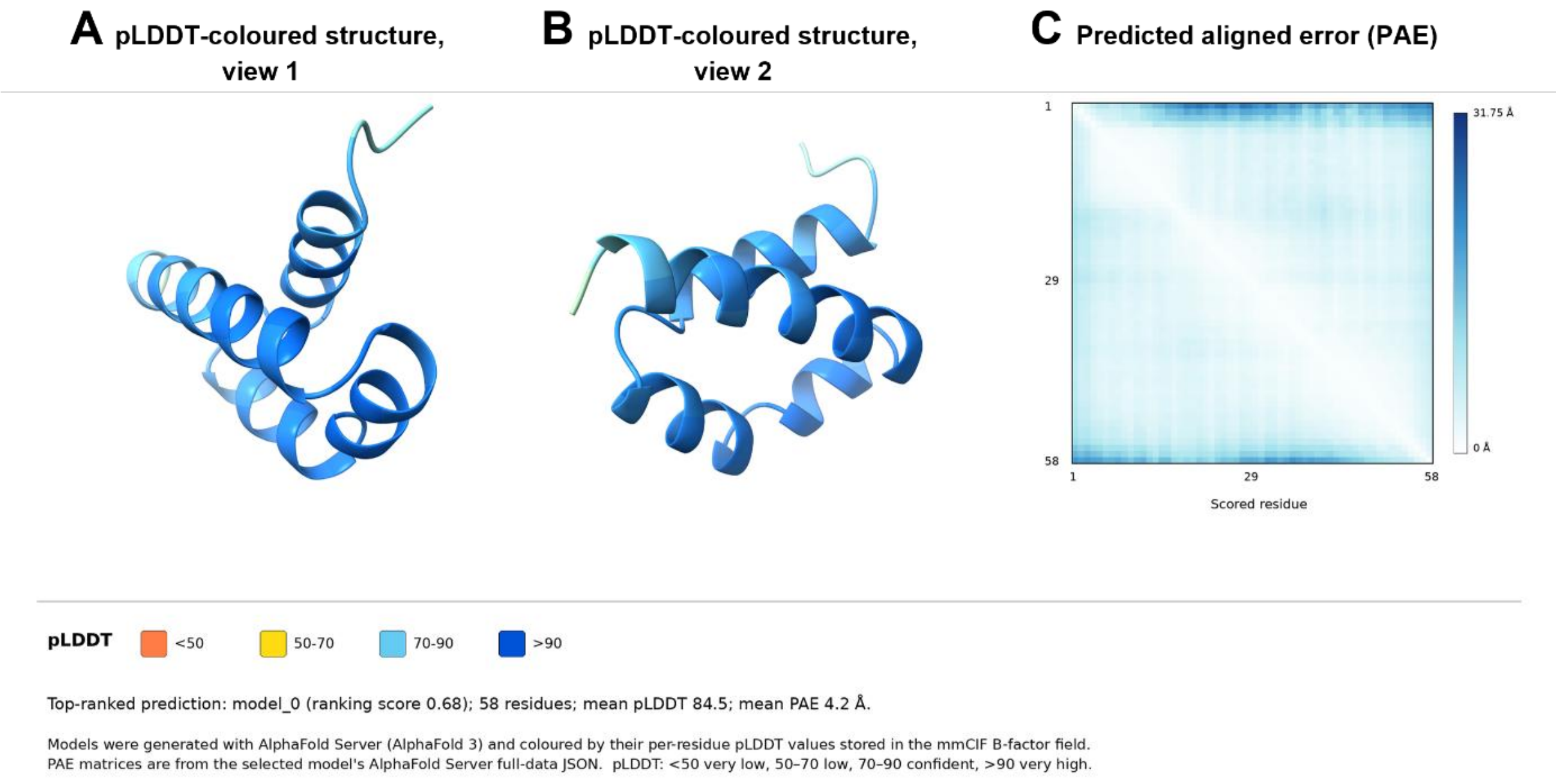

Supplementary Figure 13. AlphaFold confidence assessment for CD3368.2. (A, B) Two orthogonal cartoon views of the top-ranked AlphaFold 3 prediction, coloured by pLDDT: dark blue, >90; light blue, 70–90; yellow, 50–70; orange, <50. **(C)** PAE matrix for the same prediction; lower values indicate greater confidence in the relative placement of residue pairs. The black divider in the homodimer PAE plot delineates chains A and B. The selected candidate index, ranking score, mean pLDDT and mean PAE are listed in Supplementary Table S1.

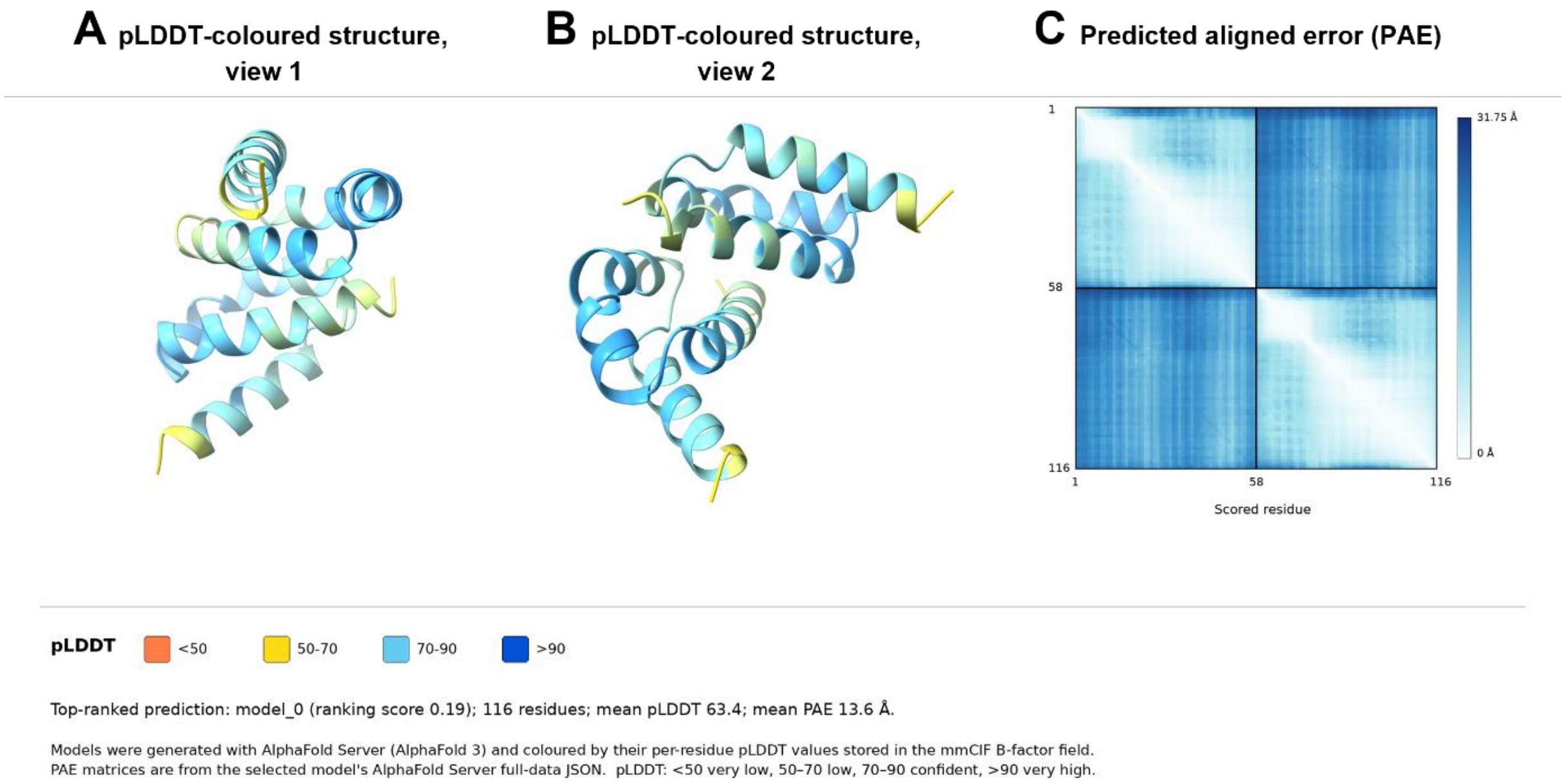

Supplementary Figure 14. AlphaFold confidence assessment for CD2309 homodimer. **(A, B)** Two orthogonal cartoon views of the top-ranked AlphaFold 3 prediction, coloured by pLDDT: dark blue, >90; light blue, 70–90; yellow, 50–70; orange, <50. **(C)** PAE matrix for the same prediction; lower values indicate greater confidence in the relative placement of residue pairs. The black divider in the homodimer PAE plot delineates chains A and B. The selected candidate index, ranking score, mean pLDDT and mean PAE are listed in Supplementary Table S1.

Supplementary Table 1. Strains and plasmids used in this study.

Strain	Genotype	Origin
E. coli		
NEB-10 beta	Δ*(ara-leu) 7697 araD139 fhuA* Δ*lacX74 galK16 galE15 e14-* ϕ*80*d*lacZ*Δ*M15 recA1 relA1 endA1 nupG rpsL* (Str^R) *rph spoT1* Δ*(mrr-hsdRMS-mcrBC)*	New England Biolabs
HB101 (RP4)	*supE44 aa14 galK2 lacY1* Δ(*gpt-proA*) 62 *rpsL20 (*Str^R*)xyl-5 mtl-1 recA13* Δ(*mcrC-mrr*) *hsdS*$_B$ (r_B-m_B-) RP4 (Tra^+ IncP Ap^R Km^R Tc^R)	Laboratory stock
C. difficile		
630Δ*erm*	630 Δ*ermB*	Laboratory stock [2]
CDIP369 (630/p)	630 Δ*erm* strain carrying the empty plasmid pDIA6103	Laboratory stock
CNRS_CD378	630Δ*erm* P_{tet}-*dccA,* chromosomally integrated ATc-inducible promoter driving *dccA* expression	This work
CDIP601	630Δ*erm* ΔCdi1_6-*CD1980.2*	This work
CDIP964	CDIP601 carrying pDIA6574 for inducible Cdi1_6-*CD1980.2::HA* expression	This work
CNRS_CD497	630Δ*erm CD2309::SPA,* chromosomally integrated SPA tag fused in frame to the 3' end of *CD2309*	This work
CNRS_CD727	CNRS_CD497 carrying pDIA6103	This work
CNRS_CD695	CNRS_CD497 carrying pDIA5987 for inducible *dccA* expression	This work
CNRS_CD449	630Δ*erm* carrying p216 (region *CD2309::phoZ* reporter fusion)	This work
CNRS_CD467	CNRS_CD378 carrying p216 (region 1 *CD2309::phoZ* reporter fusion	This work
CNRS_CD424	630Δ*erm* carrying p215 (region 2 *CD2309::phoZ* reporter fusion)	This work

CNRS_CD447	CNRS_CD378 carrying p215 (region 2 *CD2309::phoZ* reporter fusion)	This work
CNRS_CD468	630Δ*erm* carrying p230 (region 3 *CD2309::phoZ* reporter fusion)	This work
CNRS_CD470	CNRS_CD378 carrying p230 (region 3 *CD2309::phoZ* reporter fusion)	This work
CNRS_CD386	630Δ*erm* carrying p197 (*CD1980.2* promoter*::phoZ* reporter fusion)	This work
CNRS_CD443	CNRS_CD378 carrying p197 (*CD1980.2* promoter*::phoZ* reporter fusion)	This work
CNRS_CD621	630Δ*erm* carrying p283 (region Cdi1_3:: *phoZ* reporter fusion)	This work
CNRS_CD629	CNRS_CD378 carrying p283 (region Cdi1_3:: *phoZ* reporter fusion)	This work
CDIP623	CDIP601 carrying pDIA6103 (empty plasmid)	This work
CDIP626	CDIP601 carrying pDIA6106 (inducible Cdi1_6-*CD1980.2* expression)	This work
CNRS_CD676	630Δ*erm* carrying pDIA6106 (inducible Cdi1_6-*CD1980.2* expression)	This work
CDIP1016	CDIP601 ΔCdi1_9-*CD2309* (Δ2)	This work
CNRS_CD390	CDIP1016 ΔCdi1_8-*CD1990.3* (Δ3)	This work
CNRS_CD523	CNRS_CD390 ΔCdi1_10-*CD1424* ΔCdi1_11-*CD3368.2* (Δ5)	This work
CNRS_CD551	CNRS_CD523 Δ*CD2386.1* Δ*CD1511.1* (Δ7)	This work
CNRS_CD643	CNRS_CD551 (Δ7) carrying pDIA5987 for inducible *dccA* expression	This work
CNRS_CD653	CNRS_CD551 (Δ7) carrying pDIA6103 (empty vector)	This work
CNRS_CD1049	CNRS_CD551 (Δ7) carrying pDIA6106 (inducible Cdi1_6-*CD1980.2* expression)	This work
CNRS_CD128	UK1	Laboratory stock[3]
CNRS_CD1100	UK1 carrying pDIA6103 (empty vector)	This work

CNRS_CD1101	UK1 carrying pDIA6106 (inducible Cdi1_6-*CD1980.2* expression)	This work
**Plasmid**		
pMSR	Allele exchange in *C. difficile* 630	This work
pDIA6103	pRPF185 Δ*gus* vector derivative	[4]
pMC358	Promoterless phoZ	[5]
pDIA6106	pDIA6103 derivative for inducible Cdi1_6-*CD1980.2* expression	This work
pDIA6574	pDIA6103 derivative for inducible Cdi1_6-*CD1980.2-HA* expression	This work
pDIA5987	pDIA6103 derivative for inducible *dccA* expression	[6]
p210	pGEMTeasy SPA-tag	Gift from E. Bentchikou
p197	pMC358 derivate for fusion of promoter region of *CD1980.2*, including the Cdi1_6 riboswitch, with *phoZ*	
p216	pMC358 derivate for fusion of promoter region 1 of *CD2309* with *phoZ*	This work
p215	pMC358 derivate for fusion of promoter region 2 of *CD2309* with *phoZ*	This work
p230	pMC358 derivate for fusion of promoter region 3 of *CD2309* with *phoZ*	This work
p283	pMC358 derivate for fusion of promoter region of *flgB*, including the Cdi1_3 riboswitch, with *phoZ*	This work

Supplementary Table 2. Oligonucleotides used in this study.

Name	Sequence (5'-3')[1]	Description
**pDIA6103 cloning**		
IMV507	GGGATTTCTCACATAAAATAGAG	5'pDIA6103 insert screening
IMV508	TAAAATAAGCTTGATCGTAGCG	3'pDIA6103 insert screening
OS499	CCCCGAGCTCTCCTATATTATGGTAATAGTAGA	5' Cdi1_6-*CD1980.2-SacI*
OS500	CGGGATCCACTCAACTGTTTACTTTATGAA	3' Cdi1_6-*CD1980.2-BamHI*
JP289	GAACATCGTATGGGTAATTATATCTGCTTACCAATACCATC	5' inverse PCR on pDIA6106 to add HA tag
JP290	CAGATTACGCTTAAAGAAATTTTATTTTCTAGAAAACC	3' inverse PCR on pDIA6106 to add HA tag
**pMSR cloning**		
JP449	CGTTTTGTAAACGAATTGC	5'pMSR insert screening
JP450	CTCACGTTAAGGGATTTTG	3'pMSR insert screening
JP012	gtttttgttaccctaagtttGGAGATAAACTATTTAATGTTGATAATTC	5' left arm ΔCdi1_6-*CD1980.2*
JP013	aatcaaattcGCTTCATTATATATTTTTCATAAAGTAAAC	3' left arm ΔCdi1_6-*CD1980.2*
JP014	taatgaagcGAATTTGATTCTTATCTAAAATGTTGG	5' right arm ΔCdi1_6-*CD1980.2*
JP015	gattatcaaaaaggagtttCCAAGTATTGCTTCTCCTATAG	3' right arm ΔCdi1_6-*CD1980.2*
JP032	GTATTATGTAAGGCTCTATACTACAATCAAG	5' ΔCdi1_6-*CD1980.2* screening
JP024	CATCAATATTCCAATTACCAATG	3' ΔCdi1_6-*CD1980.2* screening
JP307	ttttttgttaccctaagtttGTGTTTTTGTTTTTATTCATTATGG	5' left arm ΔCdi1_9-*CD2309*
JP308	ttatattaccGAACGTTAAATTATCTGAAAAAATAAAAAG	3' left arm ΔCdi1_9-*CD2309*

JP309	tttaacgttcGGTAATATAAAATGTTTAAATTTATTTTCATATTAAG	5’ right arm ΔCdi1_9-*CD2309*
JP310	agattatcaaaaaggagtttCCATTGGTTTCTTTAAACC	3’ right arm ΔCdi1_9-*CD2309*
JP314	GCCCAAATTCATTTGTAAC	5’ ΔCdi1_9-*CD2309* screening
JP312	GTGCAGAACCTGCATAGC	3’ ΔCdi1_9-*CD2309* screening
JP315	ttttttgttaccctaagtttGCCCATATTCCATCTACAC	5’ left arm ΔCdi1_8-*CD1990.3*
JP316	aatcaatgtcCAGACTAAACACGTAGAG	3’ left arm ΔCdi1_8-*CD1990.3*
JP317	gtttagtctgGACATTGATTTCTAGAAAACC	5’ right arm ΔCdi1_8-*CD1990.3*
JP318	agattatcaaaaaggagtttGTTCCAGGAAGAAACATTAAAG	3’ right arm ΔCdi1_8-*CD1990.3*
JP319	GTAACCAACCTTTGTCATAATTACTC	5’ ΔCdi1_8-*CD1990.3* screening
JP320	GCTTCTCTGTCCATCTCTTC	3’ ΔCdi1_8-*CD1990.3* screening
JP425	ttttttgttaccctaagtttCCATCATATATTGATTCAATTTGAATATATAG	5’ left arm ΔCdi1_10-*CD1424*
JP426	gggagattagCACTAGGATAAGTTATTGTTCAAC	3’ left arm ΔCdi1_10-*CD1424*
JP427	tatcctagtgCTAATCTCCCCTATATGCAAAG	5’ right arm ΔCdi1_10-*CD1424*
JP428	agattatcaaaaaggagtttGATTGAGTTCAATTGAAAGTG	3’ right arm ΔCdi1_10-*CD1424*
JP429	CGATAATCCTCTTATAGAAACTTTAG	5’ ΔCdi1_10-*CD1424* screening
JP430	GCTGAATTTATAAGCCATAAGG	3’ ΔCdi1_10-*CD1424* screening
JP431	ttttttgttaccctaagtttGCCCACTCACCATTTTTTC	5’ left arm ΔCdi1_11-*CD3368.2*

JP432	cttaattagcCAATAATACAATAGAATTGCTTATTATTCAATATAC	3’ left arm ΔCdi1_11-*CD3368.2*
JP433	tgtattattgGCTAATTAAGATAGATTTCTAGTATTC	5’ right arm ΔCdi1_11-*CD3368.2*
JP434	agattatcaaaaaggagtttCATATGTACCTACTGATTCC	3’ right arm ΔCdi1_11-*CD3368.2*
JP435	GTGGTATTTTGAAATTTAATGTTC	5’ ΔCdi1_11-*CD3368.2* screening
JP436	CAAATGAAGAGGTGATTTCC	3’ ΔCdi1_11-*CD3368.2* screening
JP047	cggtgtttttgttaccctaagtttCAACTAGCTGAACTTCGTTC	5’ left arm P_{tet}-*CD1420*
JP048	gatgcagaattcgTTTGTACTAGATTTTTGTTAAATTTTTTATTTTATTTAC	3’ left arm P_{tet}-*CD1420*
JP049	aatctagtacaaaCGAATTCTGCATCAAGCTAG	5’ P_{tet} module
JP050	caaaataaaatacGGAGCTCAGATCTGTTAAC	3’ P_{tet} module
JP051	agatctgagctccGTATTTTATTTTGGAGAAATTAATATGTTTAAAG	5’ right arm P_{tet}-*CD1420*
JP052	tcatgagattatcaaaaaggagtttCACAAAGGTATTTTAATACTTCATTG	3’ right arm P_{tet}-*CD1420*
JP065	GGAAGGTCGATTGCCC	5’ P_{tet}-*CD1420* screening
JP064	GAAGTGAGAAATTCCTGTTCTC	3’ P_{tet}-*CD1420* screening
ABL22	ttttttgttaccctaagtttGGTATGTACAAGATGAAAAAG	5’ left arm *CD2309::SPA*
ABL23	atcttctcttATTATACTTACTTACCAATACCATC	3’ left arm *CD2309::SPA*
ABL24	taagtataatAAGAGAAGATGGAAAAAGAATTTC	5’ SPA tag
ABL25	aatctgtctaCTACTTGTCATCGTCATC	3’ SPA tag
ABL26	tgacaagtagTAGACAGATTTTTTTCTAGAAAAC	5’ right arm *CD2309::SPA*
ABL27	agattatcaaaaaggagtttGAAAAAATCTATTTATAATGTCATTTCC	3’ right arm *CD2309::SPA*
ABL33	CAAGTGATGGTATTGGTAAGTAAG	5’ *CD2309::SPA* screening
ABL34	GTCTAAGTTTTTGTTATGTTTTATAATG	3’ *CD2309::SPA* screening
ABL78	ttttttgttaccctaagtttGGGTTTTTGTGGTTTCCTTG	5’ left arm *CD2309-spoZ*
ABL79	tacattgacgCTAAAAAACTTTCATGAAAAACAAAAAG	3’ left arm *CD2309-spoZ*

ABL80	agtttttagcGTCAATGTATGGGTAGATATG	5' RBS-*spoZ*
ABL81	aaaaaatctgGTTATTTGCCAATACCTTTATC	3' RBS-*spoZ*
ABL82	ggcaaataacCAGATTTTTTTCTAGAAAACCC	5' right arm *CD2309-spoZ*
ABL83	agattatcaaaaaggagtttCAAATTAACTTATTGTAGATTTTTTATTTTTTAAC	3' right arm *CD2309-spoZ*
ABL84	CTAAGGAGGATAAAAAATGATTCAG	5' *CD2309-spoZ* screening
ABL85	CTAAAAAGGGTTTTCTAGAAAAAAATC	3' *CD2309-spoZ* screening
**pMC358 cloning**		
JP725	CGTCAATGTATGGGTAGATATG	5' pMC358 linearization for construction of transcriptional fusions
JP726	CAACGTCGTGACTGGG	3' pMC358 linearization for construction of transcriptional and traanslational fusions
ABL01	AAGAAAAGAGCTTTGCTAGG	5' pMC358 linearization for construction of translational fusions (removal of *phoZ* RBS)
ABL02	ttttcccagtcacgacgttgGGATGTATATCAATTCCATACAAATATTG	5' Cdi1_6 region
ABL03	tatctacccatacattgacgCTAAAAAACATTCATAAAAACAAAAAG	3' Cdi1_6 region
ABL07	ttttcccagtcacgacgttggCTATAAAACTTTATATTTTAGAGGAAAAATAATATG	5' Cdi1_9 region
ABL08	tatctacccatacattgacgCTAAAAAACTTTCATGAAAAACAAAAAG	3' Cdi1_9 region 2
ABL09	tatctacccatacattgacgcGATTTAGTTTTATTCTACAGATTAATAATAC	3' Cdi1_9 region 1
ABL010	cctagcaaagctcttttcttCATAATGTATTTATCCCCCTAAAAAAC	3' Cdi1_9 region 3
ABL035	ttttcccagtcacgacgttgGTTACTCTATACAACATTTTAGAC	5' Cdi1_3 region
ABL036	tatctacccatacattgacgGTAAAATAGTAACTTTTTGAGATAATTTTTC	3' Cdi1_3 region
JP733	CTATTACGCCAGCTGGC	5'pMC358-screening
JP734	GGTAACCCCTAGCAAAGC	3'pMC358-screening
**qRT-PCR**		

polIIIF	TCCATCTATTGCAGGGTGGT	5’ *DNA pol III*
polIIIR	CCCAACTCTTCGCTAAGCAC	3’ *DNA pol III*
OS576	TGGATAAATGTACACATATGTTAACTGC	5’ *CD2309*
OS577	TGCAAGCAAGATTCCACAAC	3’ *CD2309*
JP335	GTTCACGCGATTACTGC	5’ *CD1980.2*
JP336	TCTGCTTACCAATACCATCA	3’ *CD1980.2*
OS587	GCAGTTCGTATGATTATTGTAATTTTT	5’ *CD1990.3*
OS588	CCATTACTTGGTGTAAACAAGACTC	3’ *CD1990.3*
JP343	GATGTACACATATGTTGACTGC	5’ *CD3368.2*
JP344	AAACAAGATTCTACCACATTTTC	3’ *CD3368.2*
JP337	CTATTCATTCATAGAAACTCAAATTG	5’ *CD1511.1*
JP338	CAAACCACATTCGCATAGT	3’ *CD1511.1*
JP339	GTGGATATTATTCATTTATTGACAC	5’ *CD2386.1*
JP340	CATTTACAAAGCAACATCATTATAC	3’ *CD2386.1*
JP947	GGCCAAAGTGTAATGCAAGG	5’ *CD2831*
JP948	GCATCTGGAACATCCGTTTT	3’ *CD2831*
OS580	CAGTAGTGGCAGTTCCAGCTT	5’ *pilA1*
OS581	CCAGTTTGACCATCTGGTGT	3’ *pilA1*
OS574	TTCCTAAGGGATGGGAAGGT	5’ *ppep-1*
OS575	ATTGCATCATGCCCTTTACC	3’ *ppep-1*

[1]Underlined bases indicate engineered restriction sites; lowercase bases indicate overlapping sequences

Supplementary Table 3. AlphaFold model quality metrics for protein structures.

Fig.	Model input	ranking score	residues	mean pLDDT	mean PAE (Å)	access date	UniProtKB identifier
S9	CD1424	0.68	58	84.592	4.222	18/02/2026	Q18BU9
S10	CD1980.2	0.68	58	82.584	4.072	18/02/2026	Not assigned
S11	CD1990.3	0.69	58	85.457	3.838	18/02/2026	Not assigned
S12	CD2309	0.69	58	85.830	4.183	18/02/2026	Q185I3

S13	CD3368.2	0.68	58	84.490	4.227	18/02/2026	Not assigned
S14	CD2309 homodimer	0.19	116	63.372	13.617	19/02/2026	Q185I3 (monomer sequence)

SUPPLEMENTARY REFERENCES

1 Fimlaid, K. A. *et al.* Global analysis of the sporulation pathway of *Clostridium difficile*. *PLoS genetics* **9**, e1003660 (2013). https://doi.org:10.1371/journal.pgen.1003660

2 Hussain, H. A., Roberts, A. P. & Mullany, P. Generation of an erythromycin-sensitive derivative of *Clostridium difficile* strain 630 (630Deltaerm) and demonstration that the conjugative transposon Tn916DeltaE enters the genome of this strain at multiple sites. *Journal of medical microbiology* **54**, 137-141 (2005).

3 Killgore, G. *et al.* Comparison of seven techniques for typing international epidemic strains of *Clostridium difficile*: restriction endonuclease analysis, pulsed-field gel electrophoresis, PCR-ribotyping, multilocus sequence typing, multilocus variable-number tandem-repeat analysis, amplified fragment length polymorphism, and surface layer protein A gene sequence typing. *Journal of clinical microbiology* **46**, 431-437 (2008). https://doi.org:10.1128/JCM.01484-07

4 Soutourina, O. A. *et al.* Genome-wide identification of regulatory RNAs in the human pathogen *Clostridium difficile*. *PLoS Genet* **9**, e1003493 (2013). https://doi.org:10.1371/journal.pgen.1003493

5 Edwards, A. N. *et al.* An alkaline phosphatase reporter for use in *Clostridium difficile*. *Anaerobe* **32**, 98-104 (2015). https://doi.org:10.1016/j.anaerobe.2015.01.002

6 Soutourina, O. A. *et al.* Genome-wide identification of regulatory RNAs in the human pathogen *Clostridium difficile*. *PLoS genetics* **9**, e1003493 (2013). https://doi.org:10.1371/journal.pgen.1003493
