# Supplementary material for: A family of small proteins links c-di-GMP riboswitch signaling with sporulation in Clostridioides difficile
Source: Nat Commun. 2026 Oct 4;17:11319. doi: 10.1038/s41467-026-78452-6 (PMC13724677; doi:10.1038/s41467-026-78452-6)
Supplement: Supplementary file 4 [file 41467_2026_78452_MOESM2_ESM.pdf]

Description of Additional Supplementary Files

File Name: Supplementary Data 1
Description: Expression of PtetdccA/WT RNA-seq (full dataset)

File Name: Supplementary Data 2
Description: Genes differentially expressed in the Ptet-dccA strain in comparison to the wild-type strain.

File Name: Supplementary Data 3
Description: Sporulation-related genes downregulated in the PtetdccA strain in comparison to the wild-type strain.

File Name: Supplementary Data 4
Description: Expression of ΔCdi1_6– CD1980.2/pCdi1_6– CD1980.2 vs ΔCdi1_6– CD1980.2/p - RNA-seq (full dataset).

File Name: Supplementary Data 5
Description: Genes differentially expressed in the strain ΔCdi1_6–CD1980.2 carrying the Ptet- Cdi1_6– CD1980.2 plasmid in comparison to the same strain carrying an empty plasmid.

File Name: Supplementary Data 6
Description: Sporulation-related genes upregulated in the strain ΔCdi1_6–CD1980.2 carrying the Ptet- Cdi1_6– CD1980.2 plasmid in comparison to the same strain carrying an empty plasmid.

File Name: Supplementary Data 7
Description: RNA-seq from wild-type strain grown for 16 h on 70:30 sporulation agar plates (full dataset).
